# Synthetic Approaches to Colloidal Nanocrystal Heterostructures Based on Metal and Metal-Oxide Materials

**DOI:** 10.3390/nano12101729

**Published:** 2022-05-18

**Authors:** Concetta Nobile, Pantaleo Davide Cozzoli

**Affiliations:** 1 CNR NANOTEC—Institute of Nanotechnology, UOS di Lecce, c/o Campus Ecotekne, Via Monteroni, 73100 Lecce, Italy; concetta.nobile@nanotec.cnr.it; 2 Department of Mathematics and Physics “Ennio De Giorgi”, c/o Campus Ecotekne, University of Salento, Via Monteroni, 73100 Lecce, Italy; 3 UdR INSTM di Lecce, c/o Campus Ecotekne, University of Salento, Via Arnesano, 73100 Lecce, Italy

**Keywords:** colloidal nanocrystals, heterostructure, liquid-phase epitaxy, metal oxides, transition-metals, seeded growth, surface energy, interfacial energy, core/shell, nanocrystal heteromers

## Abstract

Composite inorganic nanoarchitectures, based on combinations of distinct materials, represent advanced solid-state constructs, where coexistence and synergistic interactions among nonhomologous optical, magnetic, chemical, and catalytic properties lay a basis for the engineering of enhanced or even unconventional functionalities. Such systems thus hold relevance for both theoretical and applied nanotechnology-based research in diverse areas, spanning optics, electronics, energy management, (photo)catalysis, biomedicine, and environmental remediation. Wet-chemical colloidal synthetic techniques have now been refined to the point of allowing the fabrication of solution free-standing and easily processable multicomponent nanocrystals with sophisticated modular heterostructure, built upon a programmed spatial distribution of the crystal phase, composition, and anchored surface moieties. Such last-generation breeds of nanocrystals are thus composed of nanoscale domains of different materials, assembled controllably into core/shell or heteromer-type configurations through bonding epitaxial heterojunctions. This review offers a critical overview of achievements made in the design and synthetic elaboration of colloidal nanocrystal heterostructures based on diverse associations of transition metals (with emphasis on plasmonic metals) and transition-metal oxides. Synthetic strategies, all leveraging on the basic seed-mediated approach, are described and discussed with reference to the most credited mechanisms underpinning regioselective heteroepitaxial deposition. The unique properties and advanced applications allowed by such brand-new nanomaterials are also mentioned.

## 1. Introduction

The recognition of the size dependence of the chemical-physical behaviour of nanoscale solid materials has propelled the development of techniques for the programmable fabrication and atomic-level investigation of nanostructured systems with ad hoc designed properties and functionalities. A substantial body of the ongoing revolutionary scientific advancement across the breadth of nanoscience and nanotechnology research has been boosted by the unique class of nanomaterials, represented by colloidal inorganic nanocrystals (CNCs), functional crystalline nanoparticles (typically with at least one dimension below ~150 nm) that can entirely be constructed and processed in liquid media. CNCs are appealing for both fundamental studies and technological applications due to the high level of refinement with which their properties can be engineered through synthetic control over their crystal habit, geometry, and surface functionalities [1,2,3,4,5]. In addition, CNCs can be flexibly manipulated to construct mesoscopic materials, to enable functional processes, and underlie the operation of innovative devices in areas as diverse as optics, electronics, (photo)catalysis, energy transformation and storage, sensing, biomedicine, and environmental remediation [4,5,6,7].

Wet-chemical routes, which allow leveraging on the adjustment of composition of the growth environment and temperature to affect the thermodynamics and kinetics of CNC formation in solution, have emerged for their capability to address an ample library of semiconductor, metal, and oxide CNCs with accurately defined quality features across selected a breadth of dimensional and morphological regimes [1,2,3,4,5,8,9,10,11,12,13,14,15,16,17,18,19,20,21]. Recently, a paradigm shift in nanochemistry research has led to the development of nanostructures capable to exhibit enhanced, assorted, or unconventional physical-chemical properties for reinforced applications. Conceived as a means to overcome the limitations of homomaterial CNCs posed by their inherent compositional and geometric features, last-generation breeds of wet-chemically grown nanomaterials encompass various classes of colloidal nanocrystal heterostructures (CNHSs) exhibiting a spatially controlled profile of their chemical composition and crystallographic phase. CNHSs are sophisticate multicomponent hybrid nanocrystals that can comprise distinct nanocrystalline domains permanently assembled through small bonding (frequently epitaxial) interfaces into three-dimensional architectures, spanning from onion- to heteromer-type arrangements [4,5,22,23,24,25,26,27,28,29,30,31,32,33,34,35,36,37]. The development of CNHSs with a high degree of structural-compositional and configurational elaboration mimics an established paradigm of synthetic organic chemistry, where the creation of intricate organic molecules bearing a number of functional groups allows for achieving enhanced and/or enriched, or completely new properties and capabilities [33].

CNHSs can be expected to open up unprecedented horizons on fundamental insights and technological paradigms. First, solution free-standing, CNHSs made of connected inorganic domains, each providing unique physical and chemical properties, are attractive as hybrid material platforms with rich functionalities on which original processes and applications can be founded. These include, for example, the possibility to construct mesoscopic assemblies made of nanosize building blocks, to promote sequential chemical reactions, to install a spatially asymmetric arrangement of organic ligands or biomolecules on the selected surface facets exposed, to enable multimodal diagnostic and therapeutic techniques for biomedical purposes [4,5,22,23,24,25,26,27,28,29,30,31,32,34,35,36,37]. Moreover, CNHSs, in which neighbouring sections communicate electronically across the relevant heterojunctions, may allow synergistic exchange-coupling among nonhomologous properties, leading to modified, strengthened, or even completely unexpected features and functionalities. This represents an opportunity prohibited to any of the single material components, as well as to their unbound mixtures. For example, CNHSs based on semiconductors and/or metals frequently show anomalous optical absorption and/or emission and/or conductivity due to induced modifications in electronic structure, quantum confinement, charge-carrier dynamics, and/or induction of strong plasmon-exciton coupling [32,34,35,36]. In CNHSs that incorporate noble metals and magnetic materials, the synergistic interplay of magnetic, magneto-optical, and surface plasmon resonance features may lead to unusually altered or mutually switchable magnetic and optical properties through interesting, yet poorly understood exchange-interaction mechanisms [5,22,27,28,29,38,39,40,41,42,43,44]. Alterations in electronic structure, the introduction of heterojunctions, and the establishment of predesigned charge-carrier transfer pathways across calibrated potential profiles have been exploited to manipulate the performances of catalytically and (photo)catalytically active CNHSs [36,45,46,47,48]. These findings suggest that the creation of direct bonding connections among different nanostructured domains can effectively be exploitable as a smart route to tailor their chemical-physical behaviour and potential functionalities in modular CNHSs that embody properly designed configurations [22,23,24,25,27,28,29,32,33,36,38].

The programmable chemical synthesis of CNHSs with inequivalent topologies requires achieving an elevated degree of ingenuity in architectural sophistication via refined tools for control of reaction pathways. Indeed, multimaterial CNHSs form at a delicate thermodynamic-kinetic transition boundary, at which heterogeneous nucleation, the structural and geometric evolution, and chemical and structural transformations of the relevant material domains interplay with each other. Additional complications are posed by nanoscale mechanisms, such as the facet-dependent reactivity, misfit strain insurgence, atomic diffusion, and/or exchange, which can severely affect the overall development process of a CNHS architecture, but can be controlled only indirectly and on an empirical basis for any material combination within the limits of the specific reaction conditions set. The lack of comprehensive knowledge covering all the aspects of growth makes CNHS synthesis extremely challenging.

This Review offers a thorough survey of past and recent progress made in the development of CNHSs composed of transition-metals (with emphasis on noble metals) and metal-oxide materials, interconnected into regiocontrolled configurations. The epitaxial seed-mediated approaches and the underlying growth mechanisms by which CNHSs can be constructed and trapped in nonequivalent arrangements are analytically described and analysed for a diversity of material combinations. The unique properties and functionalities that characterize such breeds of sophisticated nanostructures are also recalled.

## 2. Synthesis of Nanocrystal Heterostructures: Fundamental Concepts and Formation Mechanisms

### 2.1. Synthesis of Single-Material Nanocrystals

CNCs nucleate and grow upon the reaction of precursor molecules or metal complexes in a solution environment that is usually composed of selected solvents and stabilizers, such as ligands, polymers, surfactants, catalyst additives, or soft self-assembled templates (e.g., micellar aggregates). The synthesis is triggered at a sufficiently elevated temperature, at which reactive species, conventionally termed the “monomers”, evolve and, once a critical supersaturation limit is surpassed, congregate into a condensed phase, thus sustaining the nucleation of crystalline embryos and feeding their subsequent development. The organic ligands, surfactants, and/or other coordinating agents in the reaction environment play critical functions in the process of CNC formation. They can (i) modulate the relative supersaturation degree of the solution upon complexing with the monomers, thereby affecting the chemical potential of the latter; (ii) reversibly attach onto/detach from unpassivated atoms at the surface of the evolving CNCs, which minimizes the occurrence of random coalescence events; (iii) allow gradual addition of monomers necessary for growth continuation; and (iv) serve as size- and shape-directing agents [4,5,8,9,10,11,13].

Careful regulation of temperature, the concentration ratio of precursors, and stabilizing agents allows acting on the fundamental microscopic mechanisms that underlie CNC evolution in colloidal media, including molecular diffusion, and size dependence of the relative phase stability order, and anisotropic lattice growth [4,5,8,9,10,11]. A wealth of experimental evidence and theoretical insights has suggested that the key to producing monodisperse CNCs resides in coupling a rather short nucleation burst with a temporally distinct diffusion-limited growth process [12,13,49]. Accurate size modulation and narrowing of size variance can be realized by adjusting the relative extent of monomer depletion across the nucleation and the growth phases. Depending on the particular material, a balanced dynamics may be achieved by applying ad hoc reactant injection techniques (e.g., an initial rapid “hot-injection” of reactants, followed by slow secondary additions), by leveraging on the inherent reactivity of the system (e.g., a delayed nucleation, followed by self-catalytic growth), or by promoting Ostwald ripening to facilitate size focusing of the largest nanocrystals at the expense of the dissolution of the smallest, unstable ones [12,13,49,50]. In addition, the dynamic coordination of surfactants or ligands to the surface of the growing CNCs can severely influence the structural stability of the enclosing surface facets, thereby driving lattice evolution to non-spherical (e.g., cubic, polyhedral, elongated, branched) shapes. In particular, growth-symmetry breaking at the basis of anisotropic lattice development and splitting commonly occurs for materials that form in low-symmetry crystal structures and/or manifest facile polytypism, especially under kinetically driven growth conditions (for example, under excess monomer supply, in the presence of catalysts, under confinement within soft organic lamellar templates, across crystallographically oriented attachment events, and/or under the action of applied electric or magnetic fields) [4,5,8,9,10,11,19,20,21,51,52,53,54,55].

### 2.2. Growth Thermodynamics of a Nanocrystal Heterostructure

The construction of a CNHS may involve consecutive nucleation-growth steps, across which a preformed single-component nanocrystal or multi-component heteronanocrystal (henceforth, referred to as the “substrate”) is equipped with additional domains of one or more different materials and/or subjected to partial structural/chemical transformations in a liquid environment. Depending on the conditions, each heterostructuring step can thus proceed via a specific mechanism, such as epitaxial deposition, partial red-ox or ion-exchange conversion, phase segregation, and induced fusion. The attainment of permanently connected heterojunctions between chemically and structurally dissimilar lattices can be formally regarded as the result of a process along which otherwise isolated nanocrystal domains with distinct crystal phases and compositions are induced to bond with each other upon elimination of a corresponding fraction of their exposed surfaces.

The Gibbs free energy of growth, $\Delta G_{growth}$, that accompanies the formation of a new material domain (2) over a nanocrystal substrate (1) can be evaluated as the sum of two contributions [2,5,8,9,10,11,12,13,27,28,29,54,55,56,57,58,59]:

(1) $$\Delta G_{growth}=\Delta G_{V}+\Delta G_{S}$$

where:

(i) $\Delta G_{V}=n\Delta \mu$ is the volume free energy earned ($\Delta G_{V}<0$) in the formation of the new crystalline domain upon the incorporation of n moles of the relevant monomer constituents previously existing in the original liquid phase, and Δμ is the difference in chemical potential between the monomer species in the lattice and those in the solution environment.
(ii) $\Delta G_{S}=-A_{1}\gamma_{1}+A_{2}\gamma_{2}+A_{1,2}\gamma_{1,2}$ is the total surface-interface energy cost ($\Delta G_{S}>0)$ required for:
  - the creation of the *solid*/*solution heterointerface* of area $A_{2}$ and specific interfacial energy (also simply referred to as surface energy) $\gamma_{2}$, associated with the solid surface of the newly deposited material (2) exposed to the solution medium;
  - the creation of the *solid*/*solid heterointerface* of area $A_{1,2}$ and specific interfacial energy (or simply, interface energy)$\gamma_{1,2}$, between the substrate (1) and the new material (2);
  - the elimination of a corresponding *solid*/*solution heterointerface*) of area $A_{2}$ (with $A_{2}=A_{1,2}$) and specific interfacial energy (surface energy) $\gamma_{1_{}}^{}$ from the solution-exposed solid surface of the substrate (1).

Under the assumption that the above parameters are negligibly influenced by the deposition process itself, $\gamma_{1_{}}^{}$ and $\gamma_{2}$ are dictated by the structural and geometric features of all facets in contact with the liquid medium and by the dynamic coordination of molecular species (e.g., surfactants, ligands, precursors, monomers present in the reaction environments) thereon; on the other side, $\gamma_{1,2}$ will heavily depend on the bonding strength and coincidence relationships of the adjoining lattices at the relevant heterointerface(s).

Under surface-tension (force) equilibrium conditions, the contact angle, *θ*, setting at the three-boundary region intervening among the seed (1), the heterogeneously nucleated domain of the new material (2), and the liquid solution, expressed as [2,28,29,56,59]:

(2) $$\gamma_{1}=\gamma_{2}\cos\theta +\gamma_{1,2}$$

describes the extent to which (2) “wets” (1). From Equation (2), it is evident that *θ* is dictated by the extension and atomic arrangement of the surfaces exposed by the concerned materials (through $\gamma_{1}$ and $\gamma_{2}$) and those of the connecting heterointerface (through $\gamma_{1,2}$).

From Equation (2), it follows that the growth mode of a domain of a new material (2) over a pre-existing nanocrystal substrate (1) can be predicted qualitatively based on the sign of the specific surface-interface energy change function, $\Delta \gamma_{S}$, defined as [28,29,56]:

(3) $$\Delta \gamma_{S}=\gamma_{1}-\gamma_{2}+\gamma_{1,2}\;$$

**Scheme 1:**
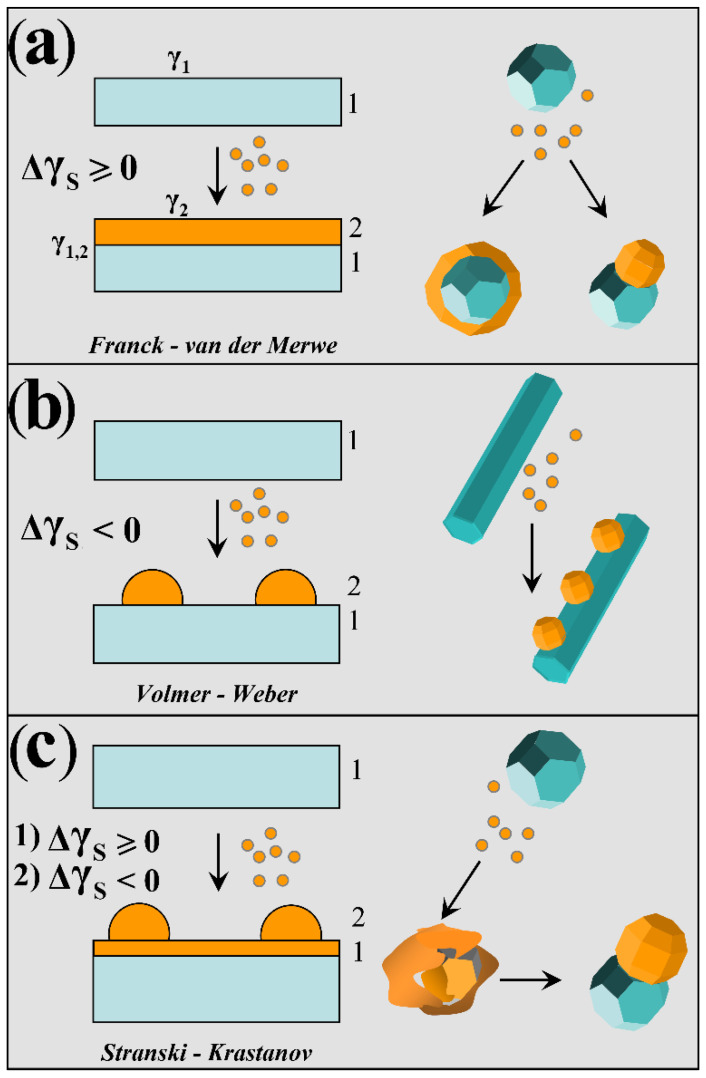
Sketch of the predictable modes by which a secondary material (labelled as “2”) may develop over a pre-existing (hetero)nanocrystal substrate (seed) made of a different primary material (labelled as as “1”): (**a**) *Franck-van der Merwe*; (**b**) *Volmer-Weber*; and (**c**) *Stranski-Krastanov* regimes (adapted with permission from Ref. [27], Copyright 2008, Wiley-VCH Verlag GmbH & Co. KGaA).

The possible growth pathways are illustrated in Scheme 1. If the joining surface of the secondary material (2) features lower energy than that of the substrate ($\gamma_{2}^{}<\gamma_{1}$) and/or is crystallographically matched with it to a large extent (hence, $\gamma_{1,2}{}_{}$ is low), then the deposition will likely proceed layer-by-layer (*θ = 0*), leading to a ubiquitous and uniform coverage. In such a case, $\Delta \gamma_{S}\geq 0$ (*Frank-van der Merwe* growth mode in Scheme 1a). By contrast, if the depositing material is characterized by higher surface energy ($\gamma_{2}^{}>\gamma_{1}$) and/or if it is remarkably lattice-mismatched (hence, $\gamma_{1,2}{}_{}$ is high), then the material will accommodate as an array of island-to-droplet-shaped domains (*θ > 0*), to minimize the overall misfit strain emerging at the heterointerfaces. Under such circumstances, $\Delta \gamma_{S}<0$ (*Volmer-Weber* growth mode in Scheme 1b). On average, the inter-domain distance can be expected to approximately scale with the amount of strain that needs to be alleviated. A hybrid-mode deposition regime could also set in if interfacial strain increases significantly during the deposition process (*Stranski-Krastinov* growth mode in Scheme 1c). In the latter case, the secondary material may evolve layer-by-layer in the early stages ($\Delta \gamma_{S}\geq 0$); then, as the depositing layer approaches a critical thickness and/or modified composition (for example, as a consequence of a chemical reaction with the substrate underneath or of a thermally induced transformation), continued growth will produce segregated domains protruding out of the initially deposited layer ($\Delta \gamma_{S}<0$) in response to accentuation of misfit strain fields. When excess strain can no longer be tolerated and, furthermore, the secondary material is characterized by strong cohesive energy, the initially deposited thin layer may tend to de-wet, allowing the exclusive evolution of discrete island-like domains in the later deposition stages [57,58].

### 2.3. Liquid-Phase Epitaxy via Seeded Growth

The most effective and commonly used strategy to prepare CNHSs builds upon the ‘*seeded growth*’ paradigm, which can be viewed as the colloidal variant of the vapour-e/liquid-phase heteroepitaxy approach. In this scheme, the growth environment is a liquid medium that contains earlier formed or pre-synthesized CNCs of a suitable material, which serve as primary seeds onto which additional domains of different secondary materials can be installed upon the reaction of the respective molecular precursors. Seeded growth leverages on a fundamental principle of the Classical Nucleation Theory (CNT) [2,5,8,9,10,11,12,13,27,28,29], according to which the activation energy barrier, $\Delta G_{het}^{*}$, that has to be surpassed to trigger heterogeneous nucleation of a secondary material onto a pre-existing condensed phase (the seeds, in the present context) is smaller than the activation energy, $\Delta G_{\mathrm{hom}}^{*}$, which would correspondingly be required to achieve homogeneous nucleation of separate crystal embryos, according to Equation (4):

(4) $$\Delta G_{het}^{*}=f(\theta )\Delta G_{\mathrm{hom}}^{*}$$

where the factor $f\left(\theta \right)$ ($0<f\left(\theta \right)<1$), termed the “wetting” function, is defined as:

(5) $$f\left(\theta \right)=\frac{\left(2+\cos\theta \right){\left(1-\cos\theta \right)}^{2}}{4}$$

and *θ* represents the contact angle described before [2,28,29,56,59].

On the other side, the activation energy barrier for the continued growth of a heterogeneously derived nucleus, $\Delta G_{growth}^{*}$, is far smaller than both $\Delta G_{\mathrm{hom}}^{*}$ and $\Delta G_{het}^{*}$ and corresponds to the limiting case of full coverage of the available seed surface ($f\left(\theta \right)\to 0$ for θ→0). In an equivalent perspective, it can be considered that induction of heterogeneous nucleation requires a substantially reduced chemical potential for the active solution monomers, compared to homogenous nucleation:

(6) $$\Delta \mu_{het}^{}<\Delta \mu_{\mathrm{hom}}^{}$$

The fundamental growth modes predicted by Equation (3) can be observed during the formation of a CNHS in a seeded-growth synthesis. In a solution medium, several mechanisms may easily allow the surface and interfacial energy terms (Equation (3)) to interplay with each other, deciding the preference for a given heterostructure architecture upon adjustment of a few reaction parameters. For example, if the conditions for the *Frank-van der Merwe* regime are satisfied for all exposed facets of a starting CNC seed, or selectively for some of them, the deposition of a secondary material can either result in a ubiquitous coverage of the seed (hence, the CNHSs feature a core@shell arrangement) or in a few fully segregated domains attached aside and sharing small heterointerfaces (in the latter case, the CNHSs adopt a heteromer-type configuration), (Scheme 1a). Differently, under circumstances favourable to the *Volmer-Weber* regime, selected facets of the seeds may allow accommodation of an array of (tiny) domains of the secondary material (Scheme 1b). If growth enters the *Stranski-Krastinov* or *de-wetting* regime, a configurational transition is likely to occur from an initial unstable architecture, where a continuous thin layer of the foreign material has formed on some or all surfaces of the seed, to a heteromer-type arrangement, in which misfit strain is remarkably attenuated (Scheme 1c).

Finally, it deserves stressing that the formation of heterointerfaces in the solution can take advantage of the surface energy modulation allowed by surfactants, ligands and other solution species as these reversibly attach onto/detach from the evolving CNHSs. The parameters $\gamma_{1}$ and $\gamma_{2}^{}$ can be altered such that the detrimental effect of large $\gamma_{1,2}{}_{}$ values can be appreciably offset. Such exclusive behaviour explains the exquisite synthetic versatility of colloidal epitaxy techniques, which can, in fact, allow the synthesis of CNHSs composed of poorly structurally compatible materials.

## 3. Heterostructures with Core@Shell Configurations

The most traditional architecture in which CNHSs have been arranged is the core@shell arrangement. Such systems are centrosymmetric or eccentric onion-like constructs, in which an inner nanocrystal “core” enclosed by a “shell” made of one or more layers of different materials, the outermost of which can directly interact with the external gaseous or liquid environment. In a sense, core@shell CNHSs may be viewed as the inorganic analogues of supramolecular complexes featuring a corona-type spatial organization [33]. Semiconductors, metals, and metal oxides arranged as core@shell heterostructures share extended connecting heterojunctions, through which direct electronic contact and hybridization may result not only in modified chemical-physical properties, compared to those of the bare constituents, but also in anomalous behaviour arising from exchange interactions.

In seeded-growth routes to core@shell CNHSs (Scheme 1a), several circumstances may be unfavorable to both heterogeneous nucleation and the development of uniform and continuous shells over the targeted core materials. Depending on the case, core size, strength of bonding, and degree of interfacial strain between the core and shell material may severely impact the extension and structural quality of the shell.

Constructing a shell over small nanocrystals is generally more difficult than coating larger nanocrystals [5,8,9,10,11,28,29,35]. Tiny seeds expose multiple small-area facets and a significant density of edges/corners; below a critical seed size, local (most frequently convex) curvature may be pronounced over a large surface fraction, resulting in atoms with high chemical potential than on a corresponding flat region [8,9,10,11]. Consequently, if the chemical bonds at the heterointerface between the core and the shell materials are relatively weak and the difference in chemical potential between the solution and crystal monomers of the shell is not sufficiently large, the addition of new (foreign) atoms to a tiny seed may be thermodynamically disfavored, preventing extended coverages. Furthermore, if the core and shell share poor similarity as to what concerns structural symmetry and/or lattice parameters, heterointerfacial mismatch can obstacle epitaxy in shell deposition, and/or the eventual relaxation of any excess misfit strain can result in the formation of detrimental defects (e.g., stacking faults, dislocations). On the other side, epitaxial overgrowth of a uniform shell can be expected to be facilitated over larger nanocrystal seeds (which indeed offer extended facets and generally low curvature), provided that misfit strain is not prohibitive, and the seeds exhibit homogeneous reactivity across all accessible surface sites/regions. Overall, the above thermodynamic arguments suggest that, even in circumstances where the chemical-potential driving force for the deposition of the secondary material is modest (the $\Delta G_{V}$ term in Equation (1) is not largely negative), and/or the heterogeneous deposition process is hindered by structural-geometric constraints or the chemical affinity between the materials is poor, a continuous shell may be successfully built over a nanocrystal core (of even small size) if the coating process is accompanied by a substantial decrease in the global surface energy (hence, of the $\Delta G_{S}$ term in Equation (1)) of the system [5,8,9,10,11,28,29].

Interestingly, in several cases, the prerequisite of lattice congruence may not necessarily impose severe restrictions on heterostructuring [5,28,29,35]. For example, when the shell deposition can occur only non-epitaxially, as in the case of shell materials with polycrystalline or even partially amorphous nature, interfacial mismatch strain may be allowed to relax plastically. Restrictions on shell growth imposed by misfit strain could be bypassed if growth is dominated by kinetic factors, rather than by thermodynamic conditions, yet at the cost of increasing the amount of crystal defects in the vicinity of the heterojunction s and/or within the shell section. In such cases, the overall core/shell heterointerface is likely to feature epitaxial connectivity at junctions of limited extension, with the crystallographic relationships between the concerned lattices varying on a local basis. In other circumstances, the presence of surfactant/ligand molecules dynamically binding to the outer shell facets may allow any interfacial energy excess to be compensated for by a consistent decrease in surface energy. Eventually, the degree of structural perfection of the shell and of the intervening heterointerfaces will heavily be reflected in the quality of the physical properties exhibited by the resulting core@shell heterostructures.

**Scheme 2:**
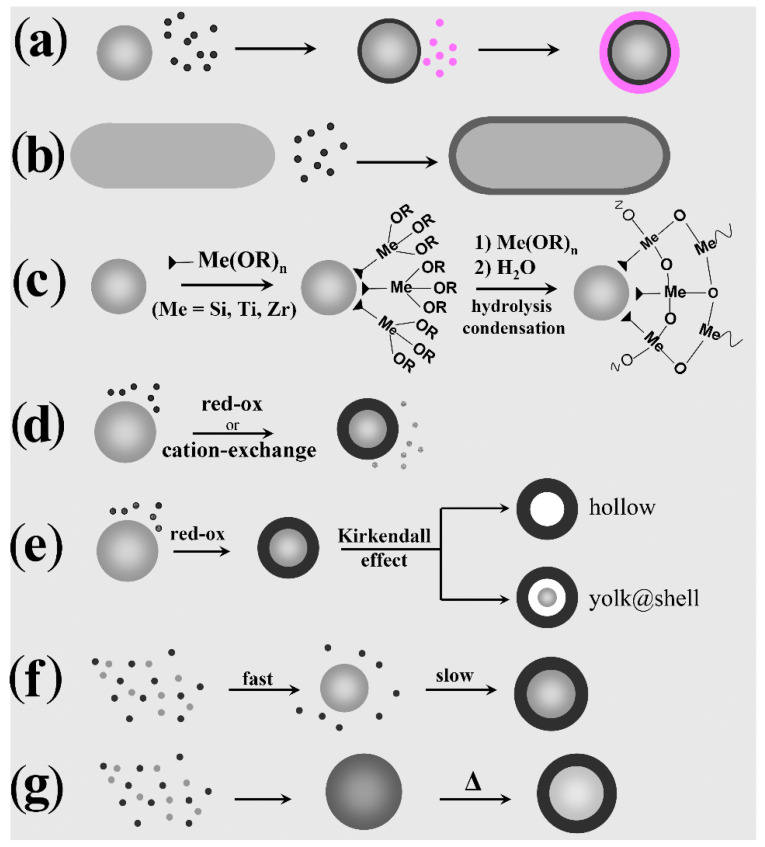
Sketch of the possible mechanisms that may allow the formation of core@shell CNHSs: (**a**,**b**) direct heterogeneous nucleation-growth of the shell material onto preformed nanocrystal seeds with defined shape and crystal phase; (**c**) silica shell growth, obtained by a priming of the seed surface, followed by silica polymerization; (**d**) direct red-ox replacement or ion-exchange reactions across the outer seed layer; (**e**) surface-confined red-ox reaction, followed by hollowing via the Kirkendall effect; (**f**) self-controlled nucleation-growth; (**g**) solid-state diffusion and thermally driven crystal-phase segregation (adapted from with permission Ref. [27], Copyright 2008, Wiley-VCH Verlag GmbH & Co. KGaA).

As of today, colloidal seeded-growth approaches have demonstrated to enable facile access to a broad diversity of structurally refined core@shell CNHSs featuring a high level of structural refinement. In the following, the synthetic schemes documented so far are classified and illustrated with reference to the particular mechanisms guiding shell formation, as summarized in Scheme 2: (a,b) direct heterogeneous nucleation-growth on tailored seeds; (c) coating with silica, achieved by polymerization over nanocrystal seeds preliminarily functionalized with primer molecules; (d,e) partial cation-exchange and/or red-ox replacement conversions performed on reactive seeds, which undergo dimensional and morphological changes; (f) self-regulation of the nucleation and growth stages; (g) solid-state diffusion and induced crystal-phase separation.

### 3.1. Direct Heterogeneous Deposition

Leveraging on the ample and consolidated toolkit of synthetic routes to single-material CNCs [5,28,29,32,35], an ample library of core@shell CNHSs based on diverse associations of metal, semiconductors, and metal oxides has been constructed by implementing the heterogeneous nucleation-growth mechanism into seeding techniques. These routes commonly realize selective deposition and growth of foreign material layers onto pre-synthesized CNC seeds (which will constitute the cores in the final core@shell heterostructure) in a suitable liquid medium, while undesired homogeneous nucleation of separate nanocrystals made of the shell material remains minimized or totally hampered (Scheme 2a,b).

Core@shell heterostructures, in which relatively extended connecting junctions between the core and the shell sections need to be attained and stabilized, are accessible when the combined materials share the same crystal structure and/or feature close lattice parameters allowing regular matching at the coincident heterointerfaces. High structural similarity is believed to be a prerequisite to promoting epitaxial deposition of a protecting shell capable to passivate dangling bonds on the surface of the seeds without triggering the formation defects. However, colloidal epitaxy routes admit case exceptions, if specific kinetic conditions and/or strain-relieving mechanisms intervene to alleviate prohibitive growth restrictions imposed by interfacial misfit strain [5,28,29,32].

Among the main goals of synthetic routes to core@shell CNHSs are control over the composition, component-phase distribution, and geometry of the seeds, and precise regulation of the thickness and the outer morphological profile of the shell domain. Effective techniques to meet these purposes rely on the careful selection of molecular precursors and organic stabilizers for the shell materials, and on calibrating their rate of injection to the seed-loaded medium.

Experimental synthesis parameters are empirically sought after on the basis of the actual reactivity and structural (in)stability manifested by the seeds in the colloidal medium chosen for the growth of the shell material. The growth temperature can eventually make a profound impact on the temporal variation of the solution supersaturation, in turn deciding whether the shell will evolve under thermodynamic or kinetic control.

**Figure 1:**
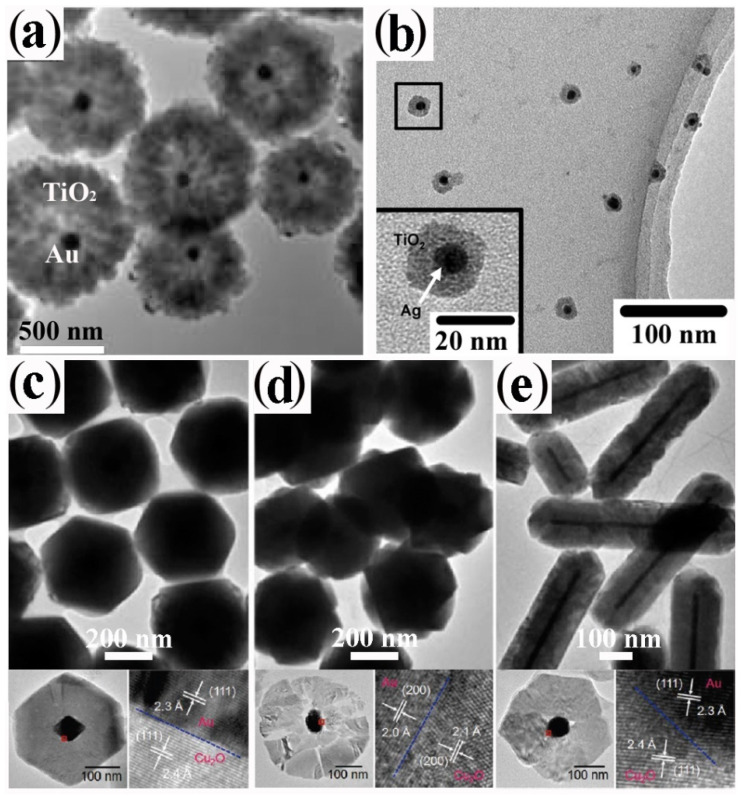
Gallery of representative core@shell CNHSs based on combinations of nonmagnetic transition-metal oxides, produced by direct single- or multiple-step heterogeneous nucleation and growth of the shell onto pre-synthesized nanocrystal seeds (that will represent the cores in the final heterostructures) (cf. Scheme 2a,b). The panels report low-magnification TEM overviews and HRTEM images of: (**a**) Au@TiO_2_ nanoreactors with inner void space (adapted with permission from Ref. [62], Copyright Wiley-VCH Verlag GmbH & Co. KGaA). (**b**) Ag@TiO_2_ CNHSs (adapted from with permission Ref. [63], Copyright American Chemical Society); (**c**–**e**) Au@Cu_2_O CNHSs with different core and shell shapes (the smaller bottom panels show cross-sectional views of the relevant interfacial regions (reproduced with permission from Ref. [65], Copyright 2009, American Chemical Society).

**Figure 2:**
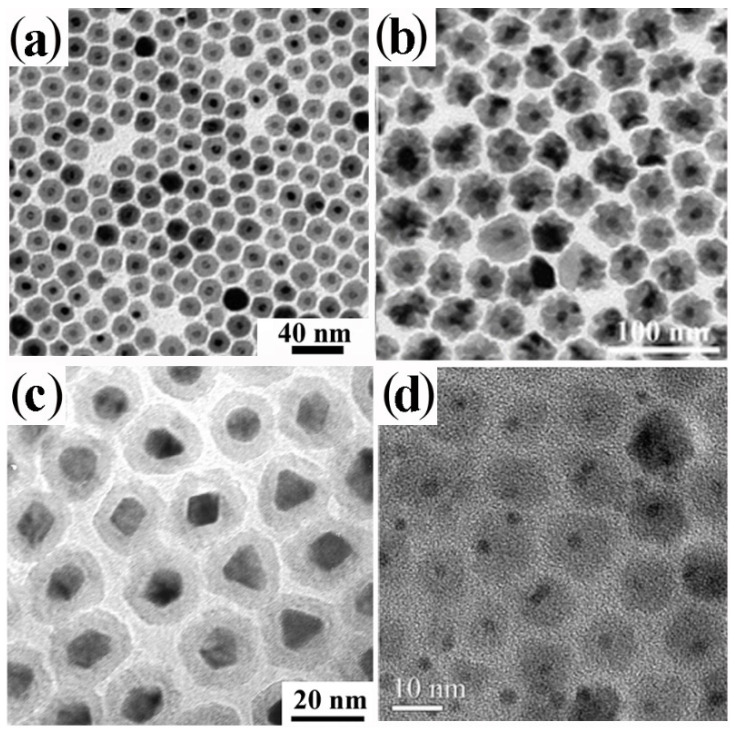
Gallery of representative core@shell CNHSs, prepared by direct heterogeneous nucleation and growth of a shell of a different material (cf. Scheme 2a). The panels display low-magnification TEM overviews and HRTEM images of: (**a**,**b**) spherical and flower-like Au@Fe_3_O_4_ CNHSs (reproduced with permission from Ref. [99], Copyright 2006, American Chemical Society); (**c**) spherical Pt@Fe_2_O_3_ CNHSs (reproduced from with permission Ref. [105], Copyright 2005, IOP Publishing; (**d**) nearly spherical FePt@ZnO CNHSs (reproduced with permission from Ref. [95], Copyright 2010, Wiley-VCH Verlag GmbH & Co. KGaA).

**Figure 3:**
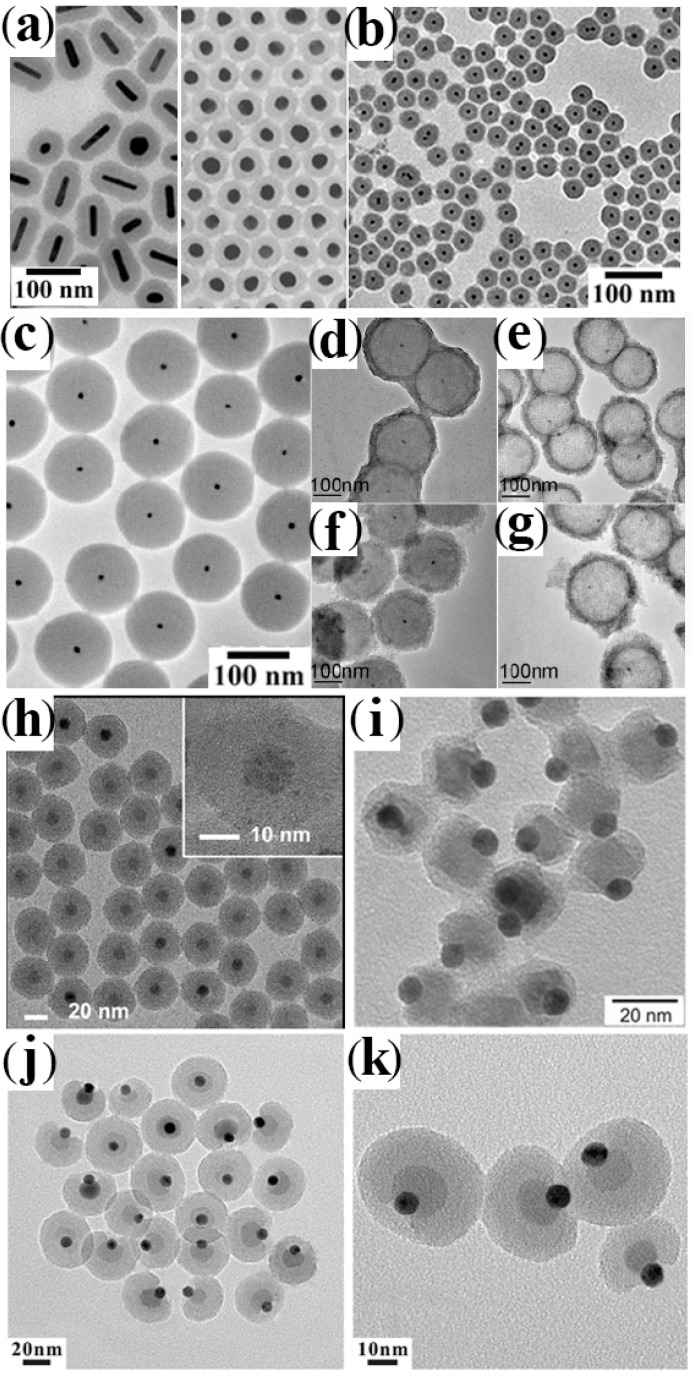
Gallery of representative core@SiO_2_-shell CNHSs synthesized after pre-activating the surface of the seed cores and then overgrowing silica (cf. Scheme 2c). The panels report TEM overviews of: (**a**) Au@SiO_2_ CNHSs with different shapes (reproduced with permission from Ref. [141], Copyright 2006, American Chemical Society); (**b**) FePt@SiO_2_ CNHs (reproduced with permission from Ref. [143], Copyright 2006, American Chemical Society); (**c**) Au@SiO_2_ core@shell CNHSs and (**d**,**e**) Au@SiO_2_@ZrO_2_ core@shell@shell CNHSs and Au@ZrO_2_ yolk@shell CNHSs thereof, respectively. (**f**,**g**) Au@SiO_2_@TiO_2_ core@shell@shell CNHSs and Au@TiO_2_ yolk-shell CNHSs thereof (adapted with permission from Ref. [134], Copyright 2009, Wiley-VCH Verlag GmbH & Co. KGaA); (**h**) ternary Au@Fe_3_O_4_@SiO_2_ CNHSs (reproduced with permission from Ref. [152], Copyright 2015, American Chemical Society); (**i**) ternary Au-MnO-heterodimer@SiO_2_ CNHSs (reproduced with permission from Ref. [153], Copyright 2014, American Chemical Society); (**j**,**k**) ternary Au–Fe_3_O_4_-heterodimer@SiO_2_ CNHSs with an asymmetric SiO_2_ shell (reproduced with permission from Ref. [161], Copyright 2014, Royal Chemical Society).

In the next paragraphs, the most significant synthetic achievements in the field are described and analysed, with emphasis on issues and drawbacks associated with the formation of core@shell heterostructures that combine metal and metal-oxide materials. An ample selection of CNHSs based on symmetric core@shell configurations, in which the shell habit conforms to the shape profile of the core underneath, has been developed across a single or multiple seeding stages (Scheme 2a,b). Yet, within this family, CNHSs that incorporate oxides materials are under-represented, compared to their counterparts based on non-oxide compounds [28,29,32,60]. Representative galleries of low-resolution transmission electron microscopy (TEM) and high-resolution TEM (HRTEM) images in Figure 1, Figure 2 and Figure 3 illustrate the level of synthetic control documented so far for these systems.

Plasmonic-metal@oxide CNHSs made of a Au, Ag, or Al core coated with either a spherical or flower-shaped shell of TiO_2_, ZrO_2,_ or SnO_2_, have been obtained by directing hydrolysis-condensation of transition-metal alkoxides over organic-capped Au or Ag CNC seeds in mixed aqueous/non-aqueous media, in micelles or microemulsions, eventually followed by high-temperature annealing to promote oxide crystallization [27,61,62,63,64] (Figure 1a,b). The shells obtained by these pathways are normally amorphous and/or extremely porous, therefore they can guarantee chemical accessibility to the noble-metal core, while allowing modulation of its plasmonic properties. Owing to these advantageous features, these CNHSs can serve not only as catalytic nanoreactors or capacitors, but also as sacrificial templates for the preparation of hollow oxide capsules upon etching of the metal core [27]. Valuable prototypes of Au@Cu_2_O [65,66,67] and Au@CeO_2_ [68] CNHSs were also delivered by aqueous routes. Shape-tailored Au@Cu_2_O CNHSs were synthesized by a room-temperature alkaline CuCl_2_ reduction with NH_2_OH on shaped Au CNC seeds in the presence of sodium dodecylsulphate as a surfactant [65]. Regardless of the appreciable differences between the lattice parameters of Au and Cu_2_O, the as-derived core@shell CNHSs incorporated epitaxial interfaces: the shell could be tailored either with a profile conformal to that of the Au core or shaped independently over a broad size range [65] (Figure 1c–e). More recently, the formation of near-spherical Au@Cu_2_O CNHs with a mesoporous Cu_2_O shell was achieved by reduction of copper acetylacetonate over Au seeds in the presence of ascorbic acid as reductant and of *n*-oleyl-1,3-propanediamine as capping agent at 100 °C [66] A conceptually similar approach was utilized to produce electrocatalytically active spherical Au@Cu_2_O CNHSs provided with thin Cu_2_O shell [67]. In the synthesis of Au@CeO_2_ CNHs [68], a CeO_2_ shell with an adjustable degree of continuity could be deposited over Au seeds, starting from Ce(NO_3_)_2_ in an aqueous alkaline medium: the precursor was induced to hydrolyze and oxidize by dissolved O_2_ in the presence of citrate anions as a complexing agent for cerium ions and stabilizer for the evolving oxide [68]. The approach was found to be extendible to the preparation of Ag@CeO_2_ CNHs [68].

Several types of CNHSs with reverse oxide@metal core@shell configuration were synthesized by chemical [27,69,70,71,72,73] or photocatalyzed reduction [20,56,57,58,59] of metal-ion precursors on weakly ligand-protected oxide CNCs in alcohols, which led to either discontinuous or uninterrupted metal (Au, Pt, Ag or Cu) layer onto the starting ZnO or TiO_2_ seeds. These architectures have been considered as active platforms for accomplishing solar energy conversion, in which the relative positioning of the metal and semiconductor bands promotes charge-carrier separation after photoexcitation of the oxide and the surface plasmon resonances induced on the noble metal may propel the photocatalytic activity and/or enhance the electron-storing capability of the system [74,75,76,77,78] Catalytically active hollow metal nanocapsules were obtained by a generalized approach that consisted in etching the core material out of pre-synthesized metal-oxide@metal core@shell CNHSs with suitable composition and etchant-permeable shells [14,16,17,79].

A broad library of core@shell CNHSs that integrate one magnetic transition-metal oxide component has been synthesized by direct heterogeneous-nucleation approaches (Scheme 2a). Exemplary heterostructure products are shown in Figure 2.

According to the devised synthesis schemes in organic media, surfactant-stabilized Fe, Fe_3_O_4,_ or FePt CNCs were utilized as substrates for the reduction of Au(III)- or Ag(I)-salts in mixtures of oleic acid (OLAC) and/or oleyl amine (OLAM) at ~170–180 °C [80,81,82,83,84]. A moderate temperature was needed to facilitate surfactant detachment from the surface of the seeds, thereby making them more susceptible to being attacked by the available metal monomers. According to another account, OLAC/OLAM-protected Fe_3_O_4_ n CNCs and alkyl-thiol-coated Au CNCs co-loaded in toluene were stimulated to react and reconfigure at 150 °C into Fe_3_O_4_@Au CNHSs in the presence of an alkylammonium bromide as an additive promoter [85]. It is plausible that the observed evolution was driven by the associated reduction of the overall surface energy of the system, compared to that of starting mixture of Fe_3_O_4_ and Au CNCs.

The low-temperature synthesis of core@shell CNHSs has been attempted also in aqueous media. Unfortunately, in such environments, the heterostructure population is observed to develop with broad size variance and to suffer from poor colloidal stability against irreversible precipitation. A few case exceptions may be found, such as the circumstances in which Fe_x_O_y_@Au and Fe_x_O_y_@Ag CNHSs bearing thick metal shells were synthesized [85,86] These heterostructures were obtained by directing metal deposition onto weakly-passivated Fe_x_O_y_ seeds upon reduction of Au(III) salts with hydroxylamine [66] or AgNO_3_ with NaBH_4_ [85], respectively, in aqueous suspension or water-in-oil microemulsions. In another report, the preparation of Fe_3_O_4_@Au CNHSs with a star-shaped Au shell was described [87]. More recently, spherical Fe_3_O_4_@Au CNHSs with adjustable dimensions and narrow size distribution were constructed inside nanosized tri-block copolymer templates, where the core and shell domains evolved in consecutive steps [88]. Interestingly, on the basis of the results of detailed investigations performed, all magnetic@plasmonic CNHSs were assessed to exhibit surface plasmon absorption depending on shell thickness and modified magnetic parameters (e.g., reduced blocking temperature, enhanced coercivity), relative to those of the individual materials. These facts were explained by considering that the strength of interparticle coupling could depend on a screening effect stemming from the metal coverage [22,23,24,25,80,81,83,84]. These nanoheterostructures, which integrate an inner ferro- or ferri-magnetic core and a plasmonic-responsive shell easy to functionalize with biomolecules, have been put forward as key elements for a number of applications, such as in catalysis, in bioassay, and magnetically assisted bio-separation, in bacteria eradication, in biomedical diagnostics (e.g., as contrast agents for magnetic resonance imaging, MRI) and therapeutics (e.g., as photothermal agents) [22,23,24,25,80,81,83,85,87,89,90].

CNHSs based on an inverted plasmonic@magnetic arrangement were obtained by seeding with noble-metal CNCs under solvothermal [91], or nonhydrolytic sol-gel condensation reaction conditions, in which homogeneous nucleation of transition-metal oxide materials is highly kinetically hindered [13,27,92,93,94,95,96,97,98]. For example, monodisperse Au@Fe_3_O_4_ CNHSs with a shape-controlled Fe_3_O_4_ shell epitaxially joint to its Au core underneath (Figure 2a,b) were grown by reacting small Au CNCs with Fe(CO)_5_ at 200–300 °C in an octadecene (ODE) or dioctyl ether (DOE) medium containing OLAC and OLAM as colloidal stabilizers [99,100,101,102] Ag@Fe_3_O_4_ CNHSs were prepared using analogous approaches [103,104].

Regular-sized Pt@Fe_2_O_3_ CNHSs were synthesized according to one-pot, two-step synthesis schemes [96,105,106]. In alkyl carboxylic acid/amine mixtures, Pt or FePd CNCs were primarily nucleated in situ upon reduction of platinum acetylacetonate with alkyldiols, after which the preformed seeds reacted with the iron precursor allowing shell deposition [96,105,106] (Figure 2c). Unusual urchin-shaped FePd@Fe_3_O_4_ nanocomposites, made of FePd nanoparticle clusters and rod-like Fe_3_O_4_ spikes, were produced upon combining Fe(CO)_5_ decomposition with palladium(II) acetylacetonate reduction under harsh conditions [107]. These objects could be transformed into exchange-coupled L1_0_–FePd/α-Fe nanomagnets upon solid-state reductive annealing. Such treatment, during which the original Fe_3_O_4_ phase was converted to α-Fe, ultimately yielded a nanocomposite matrix made of interconnected nanoscale α-Fe and FePd grains [107]. Lately, in the context of a two-step seeded-growth approach, the high-temperature reaction of Pt seeds with iron palmitate or myristate was exploited to synthesize Pt@Fe_3_O_4_ CNHSs, in which the Pt cores are enclosed by an epitaxially connected triangular-prismatic shell [108]. Unconventional asymmetric heterostructures composed of Au nanorods unevenly decorated with α-Fe_2_O_3_ domains have additionally been developed [109].

CNHSs built on associations of magnetic and semiconductor-oxide materials have been developed. FePt@ZnO CNHSs with high shell-to-core volume ratio were prepared by OLAM-induced aminolysis of zinc acetate in FePt seed/DOE media at 260 °C [95]. These heterostructures exhibited the superparamagnetic behaviour of nanoscale FePt and the fluorescence emission of ZnO, a valuable piezoelectric material, were envisioned to serve for realizing future magnetically controlled electromechanical devices (Figure 2d). Another account reported on the synthesis of magneto-opto-fluorescent FeAu@ZnO CNHSs, capped with poly(ethylene glycol)-block-poly(propylene glycol)-block-poly(ethylene glycol), which resulted in being dispersible in solvents of broadly varying polarity [110]. Finally, the recent demonstration of unconventional rattle-type Fe_3_O_4_@CuS CNHSs deserves to be mentioned [111]. These nanoheterostructures, which entailed covellite CuS, a heavily self-doped semiconductor supporting surface plasmon resonance properties [112], were proven to be exploitable as magnetically driven photoinduced hyperthermia agents across the near-infrared windows of biological relevance, and for MRI-based detection of cancer cells [111].

Pyrolysis of metal acetates at 320 °C in a hot noncoordinating solvent (DOE or ODE) loaded with tri-*n*-octylamine and OLAC was manipulated to deposit a shell of antiferromagnetic (AFM) CoO, MnO, or NiO onto monodisperse ferromagnetic (FM) or ferrimagnetic (FiM) seeds of metal alloys and inverse-spinel metal ferrites [113,114,115,116,117,118,119,120,121]. The resulting bi-magnetic CNHSs manifested the unequivocal fingerprint of magnetic exchange coupling at the heterojunctions between the FM (or FiM) and AFM domains, which led to increased unidirectional anisotropy. In several cases, anomalous exchange-bias effects resulted in enhanced thermal stability of the magnetization, a property that renders such bi-magnetic CNHSs potentially exploitable as active elements in high-density magnetic recording [38,122], as hyperthermia agents against cancer cells [117,118] and as magnetically recoverable catalysts [115]. Unique cases that are worthy to be highlighted include *fct*-FePt@MgO nanocomposites obtained upon reductive annealing of colloidal *fcc*-FePt@Fe_3_O_4_ CNHSs coated with a MgO [123], and CoFe@Fe_3_O_4_ CNHSs that were unexpectedly obtained upon Fe_3_O_4_ overgrowth over CoFe_2_O_4_ seeds [93]. Bi-magnetic FePt@MeFe_2_O_4_ CNHSs (Me = Fe, Co) with a coherent core/shell heterointerface were produced by a two-step synthetic scheme [93,97,98]. In the proposed procedure, Fe_x_Pt_1−x_ seeds, obtained by simultaneous platinum acetylacetonate reduction and Fe(CO)_5_ decomposition, were covered with an MFe_2_O_4_ shell upon co-decomposition of cobalt and iron acetylacetonate in the presence of OLAC and OLAM. The geometric parameters of the CNHSs could be tailored by regulation of the nominal concentration ratio of the reactants, while the seed content set the relative extent of shell precursor depletion between the heterogeneous nucleation and growth stages, respectively. Magnetic measurements disclosed strong exchange interactions between the interfaced magnetic phases, suggesting that their volume proportions determined the ultimate coercivity of the heterostructures.

Upon implementing slightly modified protocols for shell growth, several materials could be conveniently combined into functional core@shell heterostructures. Among others, Co@Fe_2_O_3_ and SmCo_5.2_@Fe_2_O_3_ CNHSs (Me = Co, SmCo_5.2_), surface-modified with biomolecules, were applied to the sorting of histidine-tagged proteins [92,94]. A technologically valuable strategy that integrated a liquid-phase processing step with a solid-state reaction was developed to produce hard nanomagnets [94]. The devised technique consisted of promoting the transformation of preformed Co@Sm_2_O_3_ CNHSs to nanosized *hcp* SmCo_5_ by reductive annealing.

### 3.2. Silica Coating

Colloidal heterostructures capable to expose hydrophilic and biocompatible surfaces hold great promise for application across several fields of biomedical relevance. Silica (SiO_2_) has been universally identified as an excellent material candidate for creating such desirable types of surfaces.

Techniques of general applicability to enwrap CNCs within a SiO_2_ shell have been intensively pursued and have soon reached a high level of advancement. Most synthetic procedures introduce a “priming” step, which involves implanting a monolayer of alkoxide groups onto the surface of the CNC seeds by use of suitable coupling agents, such as polymers, gelatine, or silicon-containing organometallic compounds [100,101,102,103,104,105,106,107,108,109,110,111,112,113]. Subsequent hydrolysis and condensation reactions driven by calibrated addition of water and silicon alkoxide result in the progressive construction of a SiO_2_ lattice around the target seeds [124,125,126,127,128,129,130,131,132,133,134,135,136,137,138,139,140,141,142,143,144,145,146,147,148,149,150,151,152,153,154,155,156,157,158,159,160,161] (Scheme 2c). The environment-exposed surface of the shell can then be utilized as a ground for installing chemical moieties that would be otherwise difficult to attach directly onto the surface of the pristine CNC sees. It is worth stressing that, because of the amorphous and porous structure of the SiO_2_ lattice, which makes it tolerant to huge interface strain, the thickness of a SiO_2_ shell may greatly exceed that of conventional shells made of inorganic materials.

A variety of metal and metal-oxide CNCs have been successfully encapsulated within a SiO_2_ layer. Representative examples of such CNHSs are collected and shown in the following Figure 3.

A SiO_2_ coating on organic-capped CNCs of semiconductor and transition-metal materials, such as Au, Ag, Pt, Co, Fe and their alloys [124,126,130,131,142,143,144,145,146,147,148,149,150,151,152,153], has been found to facilitate the transfer of such nanostructures (which are originally hydrophobic) to aqueous environments of biological significance for purposes as diverse as cell targeting, labelling and/or separation, cell separation, and imaging. In such circumstances, the protecting silica shell allows minimizing the release of toxic metal ions from the core to the aqueous medium in contact with the outer shell surface (Figure 3a,b).

The enwrapping of noble-metal CNCs within a SiO_2_ shell was identified as a means of tuning their surface plasmon absorption, with relevant implications for optical sensing [124,137,141,142,154]. In magnetic-core@ SiO_2_-shell CNHSs, the dielectric and magnetic losses associated with the SiO_2_ and magnetic components, respectively, were proven to synergistically enhance the electromagnetic wave absorption properties of the system [131]. On the other side, transition-metal@SiO_2_ CNHSc have drawn attention as versatile building blocks for the construction of space-filling super-structures (e.g., inverse lattices, opals, and photonic crystals) or as miniaturized reactors, whereby molecules diffusing through the porous shell can reach the catalytically active metal domain buried inside and react at its surface in a confined nanosize space [124,132,156,157,158].

**Figure 4:**
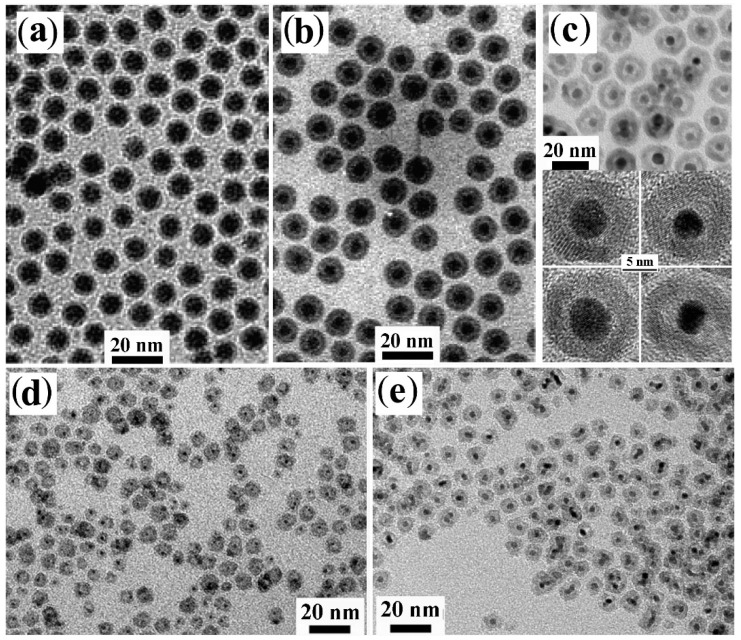
Gallery of representative core@shell and yolk@shell CNHSs prepared by red-ox replacement over on metal seeds, followed by hollowing (cf. Scheme 2d,e). The panels display low-magnification TEM and HRTEM images of: (**a**,**b**) Fe@Fe_3_O_4_ core@shell CNHSs and corresponding yolk@shell Fe@Fe_3_O_4_ CNHSs thereof, respectively (adapted with permission from Ref. [174], Copyright 2007, American Chemical Society); (**c**) Au@Fe_3_O_4_ yolk@shell CNHSs (adapted with permission from Ref. [191], Copyright 2008, Wiley-VCH Verlag GmbH & Co. KGaA); (**d**,**e**) FePt@Fe_3_O_4_ and Pt@Fe_3_O_4_ yolk@shell CNHSs (adapted with permission from Ref. [188], Copyright 2008, American Chemical Society).

Three-component core@SiO_2_ CNHSs, made of magnetic/luminescent magnetic/metal cores embedded within a SiO_2_ shell, or of metal cores covered with double-layer SiO_2_@metal-oxide shells (where the metal-oxide layer could be TiO_2_, ZrO_2_ or Y_2_O_3_) were also achieved by multiple seeding-step based approaches [133,134,135,149,159,160]. Upon selective etching of the core and/or partial leaching of the SiO_2_ interlayer, metal@SiO_2_@Me_x_O_y_ were transformed to thermally stabilized yolk@shell nanostructures (Figure 4e–i), which comprised an extremely catalytically active metal domain with the naked surface, separated by an empty space from the outer walls, composed of the original shell material [132,133,134,135,149].

Recent reports have indicated the possibility to extend the application of the SiO_2_-coating technique to enwrap even multicomponent heterostructured cores. For example, preformed Au@Fe_3_O_4_ core@shell CNHSs and Au-MnO and Au-Fe_3_O_4_ heterodimer CNHSs were used as the starting biphasic seed on which SiO_2_ overgrowth resulted in a chemically accessible centrosymmetric or eccentric shell [153,161] (Figure 3h–k). To pursue enhanced functionality, there have been efforts toward SiO_2_-protected CNHS systems integrating magnetic and fluorescent materials. One example is represented by a complex FePt@Fe_3_O_4_-CdSe@SiO_2_ system, which was constructed starting from spherical FePt@Fe_3_O_4_ core@shell CNHSs [162]. The heterostructure seeds were first decorated with tiny CdSe nanocrystal satellites and then coated with SiO_2_ shell in a reverse-micelle microemulsion to minimize solvent-induced quenching of the CdSe photoluminescence [162].

Interestingly, it has been demonstrated that SiO_2_ shells can be used as protecting confined environments inside which the trapped cores may be driven to undergo structural-compositional transformations, ultimately leading to nanostructures that would be difficult to engineer by other routes [151,152,163,164,165]. In the inner void space offered by metal@SiO_2_ yolk@shell CNHSs or across the channel network in the mesoporous SiO_2_ shell of metal@SiO_2_ core@shell CNHSs efficient catalytic reactions may be hosted and proceed with the active metal cores staying prevented from possible detrimental aggregation and/or undesired leaching [115,132,134].

### 3.3. Shell Formation by Galvanic Replacement and Transformative Reactions

A refined strategy toward core@shell CNHSs relies on the partial sacrificial transformation of an external portion of a starting CNC seed to a different material by a red-ox replacement reaction [166,167,168,169,170,171,172,173,174,175] (Scheme 2d,e). In this regard, transition-metals appear to be convenient as seed substrates owing to their susceptibility towards controllable transmetalation in liquid media [168,169,170] and towards oxidation when exposed to atmospheric or solvated O_2_, and other oxidizing agents [171,172,173,174,175,176,177,178,179,180,181,182,183,184,185,186,187,188,189,190,191,192,193,194,195,196,197,198,199]. Galvanic transformation allows the construction of core@shell CNHSs, whereby the average valence state of metal species present in the core is different from that of metal species located in the shell. Examples of such types of CNHSs are collected in Figure 4.

Several prototypes of metal@metal-oxide CNHS systems have been obtained by partial oxidation of their respective metal core seeds. Monodisperse Ni@NiO [171] and Fe@Fe_x_O_y_ CNHSs with variable-crystallinity cores [172] were formed upon exposing organic-capped Ni and Fe CNCs, respectively, to air (hence, to O_2_)_._ Adjustment of temperature and reagent proportions enabled control over the crystal-phase distributions of the cores, enabling a transition from Fe@Fe_3_O_4_ to Fe*_x_*O@Fe_3_O_4_ and Fe*_x_*O@γ-Fe_2_O_3_ core@shell CNHSs [176,177,178]. By an analogous mechanism, Ag@Ag_2_O core@shell domains were formed over solution free-standing layered double-hydroxide nanosheets [179]. Such heterostructures were utilized for diverse scopes, ranging from biomolecule tagging, and magnetic-assisted sorting, to MRI imaging and photothermal therapy [171,179,180,181,182,183]. In another study, judicious O_2_ supply to Co CNCs allowed access to ferromagnetic (FM)/antiferromagnetic (AFM) Co@CoO CNHSs, where the thickness of the CoO shell could be increased systematically, up to achieving full oxidation of the starting seeds to Co_x_O_y_ CNCs [167,173]. Alternative oxidizing agents were also successfully applied to synthesize these types of core@shell CNHSs [184].

Significant progress in combined control of shape and compositions has been demonstrated with the fabrication of elaborate yolk@shell CNHSs by the so-called Kirkendall mechanism, a process of solid-state atomic diffusion that proceeds via vacancy exchange rather than by atomic interchange [14,15,16,17,30,31]. It has indeed been documented that, in a nanoscale crystal, where the interior part of the lattice accommodates fast-migrating atomic species (e.g., metal cations) and the external portions supply slow-migrating species (e.g., oxygen anions), a net transfer of mass from the core outwards can occur, accompanied by a congregation of the generated vacancies into a single large void [14,15] (Scheme 2e). Material systems that are easily predisposed to host Kirkendall diffusion are represented by metallic nanoparticles coated with a thin meta-oxide layer. For example, upon combining an ODE solution of OLAM-capped amorphous Fe nanoparticles with a diluted O_2_ flow or with trimethylamine *N*-oxide, a systematic progression from low-crystalline Fe@Fe_x_O_y_ core@shell nanoparticles [172,185,186] to Fe@Fe_3_O_4_ yolk@shell CNHSs (Figure 4a,b), then to hollow Fe_3_O_4_ CNCs was observed [174,175]. Such unconventional topological evolution suggested that the galvanic-based reaction toolkit to CNHSs indeed holds unexplored potential for elaborating structurally sophisticated heterostructures with sub-nanometer precision. Actually, the selective induction of the Kirkendall diffusion into the shell of pre-synthesized core@shell CNHSs has been proven to be a unique strategy to construct a library of yolk@shell architectures, in which a void space separates the core and shell domains made of distinct materials. This has been demonstrated by the successful preparation of a library of yolk@shell CNHSs, such as that of Au@Fe_3_O_4_, Pt@CoO, FePt@Fe_3_O_4_, and FePt@CoS_2_ [187,188,189,190,191,192]. These systems were synthesized upon mild oxidation of Au@Fe, Pt@Co, FePt@Fe, and FePt@Co core@shell CNHS seeds with HAuCl_4_, O_2,_ or S, respectively, in organic mixtures at low temperatures [187,188,189,190,191,192]. (Figure 4c–e). The existence of an inner space within the yolk@shell constructs was probed on the basis of the outcome of chemical reactions catalyzed by the buried metal core. For instance, Pt@CoO yolk@shell CNHSs were found to exhibit the typical catalytic activity of Pt for the hydrogenation of ethylene. Dedicated experiments revealed that, because of their small sizes, the molecular reactants and byproducts could approach the surface of the buried Pt core by permeating through the porous CoO shell [189,193]. Encouraged by the success of prototypical biological assays, in which FePt@CoS_2_ and Co@Au yolk@shell CNHSs were proven to act as effective killers for cancer cells [165] and as carriers for gene delivery [187], respectively, FePt@Fe_3_O_4_ yolk@shell CNHSs were assessed for their potential as MRI contrast agents and as anticancer drugs [188].

### 3.4. Shell Transformation via Cation-Exchange Reactions

Another versatile approach to engineering both the composition and phase distribution in CNHSs, an alternative to less straightforward routes, relies on performing partial reversible transformations mediated by solid-state ion exchange [28,29,194] (Scheme 2d). In particular, calibrated metal-cation exchange reactions have been shown to allow restructuring of preformed CNCs of a given composition into alloy CNCs, or to CNHSs with core@shell, segmented, or striped profiles.

**Figure 5:**
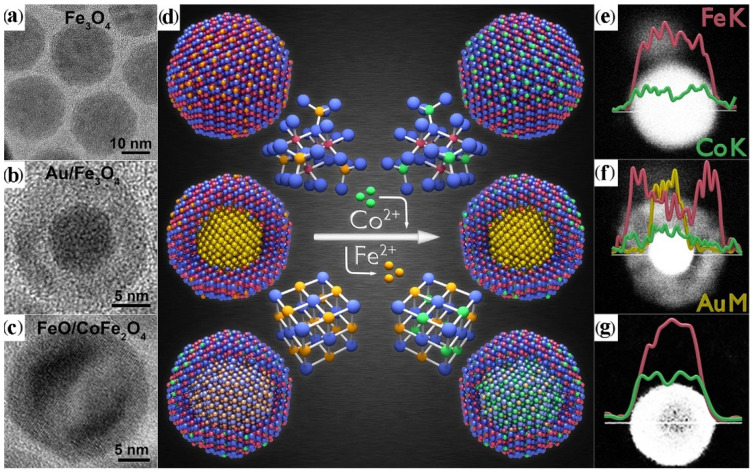
Gallery of representative core@shell CNHSs resulting from partial on-exchange reactions (cf. Scheme 2d) across preformed core@shell CNHS seeds: (**a**–**c**) TEM images of the starting substrates, namely homogeneous Fe_3_O_4_ CNCs, Au@Fe_3_O_4_ core@shell CNHSs, and Fe_1−x_O@CoFe_2_O_4_ core@shell CNHSs, respectively; (**d**) Scheme of the cation-exchange process intervening in the nanocrystal seeds shown in panels (**a**–**c**) (blue balls represent oxygen atoms; red balls, Fe^3+^; orange balls, Fe^2+^; green balls, Co^2+^; gold color, Au atoms). (**e**–**g**) High-angle annular dark-field STEM images of the nanostructures are shown in panels (**a**–**c**), respectively, after Fe^2+^ to Co^2+^ cation exchange. The distributions for Fe (red) and Co (green), and Au (golden) across the nanocrystals, shown by the EDX line scans (obtained for transitions from the K or M shell, respectively), reveal Co doping after the cation-exchange reaction (reproduced with permission from Ref. [195], Copyright 2013, American Chemical Society).

According to prior knowledge gained on metal-chalcogenide CNCs, the activation of the cation-exchange process on a nanocrystal lattice is dictated by the solvation energies of the entering and leaving cations as well as on the availability of appropriate metal-sequestering agents [28,29,194]. Unlike the galvanic replacement route illustrated previously, across which the nanocrystals may undergo morphological changes, cation-exchange reactions allow preservation of the shape of the starting CNC template because the sublattice of the associated anions (which are bulkier and may thus diffuse much more slowly, compared to the cations) is substantially conserved. Among others, core@shell CNHSs constitute an intriguing class of platforms where ion-exchange conversions may be directed with spatial selectivity to involve either the core or the shell domain. The case addressed in Figure 5 illustrates the synthetic potential of this route. Pre-synthesized OLAC-capped Fe_3_O_4_ CNCs, magneto-plasmonic Au@Fe_3_O_4_ and bi-magnetic Fe1-xO@Fe_3_O_4_ core@shell CNHSs, respectively, were induced to react with a CoCl_2_-OLAM complex in the presence of tri-n-octyl phosphine (TOP) that served as Fe^2+^ chelator at around 200 °C. The resulting products were Co_x_Fe_2−x_O_4_ CNCs, Au@Co_x_Fe_2−x_O_4,_ and Fe_1−x_Co_x_O@CoFe_2_O_4_ core@shell CNHSs, respectively, where the relevant metal-ferrite phased were homogeneous solid alloys [195]. Comparative structural-compositional and magnetic investigations of the nanostructures indicated that Co^2+^ cations had diffused and heavily doped the metal ferrite phase (Fe_3_O_4_ or CoFe_2_O_4_) of the pristine seeds (Figure 5a–g).

### 3.5. Self-Regulated Nucleation and Growth

**Figure 6:**
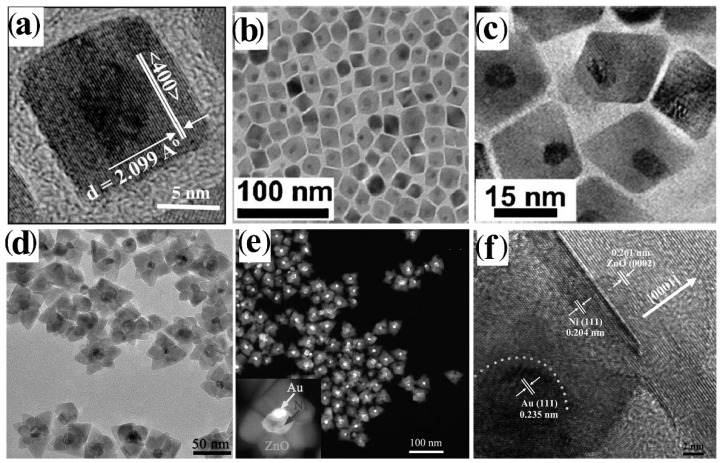
Gallery of representative core@shell CNHSs achieved by self-regulated nucleation-growth mechanisms (cf. Scheme 2f). The panels report: (**a**–**c**) TEM images of Co@Fe_2_O_3_ CNHSs with rhombohedral-shaped shells (reproduced with permission from Ref. [203], Copyright 2011, Royal Society of Chemistry). (**d**–**f**) TEM, high-angle annular dark-field STEM, and HRTEM images of Ni@Au@ZnO CNHSs composed of a flower-like multipetal ZnO shell covering asymmetric Ni@Au core@shell cores (reproduced with permission Ref. from [206], Copyright 2015, Wiley-VCH Verlag GmbH & Co. KGaA).

It has been discovered that, in certain conditions, core@shell CNHSs may also be grown by one-pot, single-step procedures without the necessity of a preliminary synthesis of the core seeds as an independent step. In such cases, in fact, the formation of the core and the shell materials occurs sequentially, yet in the same reaction environment in which all necessary reactants have been mixed. CNHS systems that have been created by such routes are discussed based on the case studies reported in Figure 6a–c.

For selected systems, the intrinsic reactivities of the molecular precursors to generate the core and shell lattices, or, equally, the kinetic barriers for the homogeneous nucleation of CNCs of either component may be differentiated such that: (i) the two materials are induced nucleate or develop at different times and/or temperatures; and (ii) the shell grows appreciably only if its heterogeneous deposition is process catalysed at the surface of preformed seeds of the other material, which have been produced in situ in a preceding step (Scheme 2f). In such cases, the standard fast “hot-injection” approach for introducing the precursors into the reaction medium is no longer necessary as a means of temporally separating the nucleation and growth processes [12,13,50]. Another practical benefit is that extra slow precursor additions to promote narrowing of the size variance along the synthesis course are normally not required [12,13,50]. Several cases that explain the concept of self-regulated nucleation/growth mechanism have been documented [196,197,198,199]. For example, biphasic Fe/Fe_x_O@Fe_3_O_4_ [198,199] and Fe_x_O@CoFe_2_O_4_ [197] CNHSs were prepared by pyrolysis of metal-oleate complexes. In the specified environments, early homogeneous nucleation and growth of Fe/Fe_x_O embryos proceeded until they approached a critical size, at which subsequent overgrowth of a Fe_3_O_4_ or CoFe_2_O_4_ layer, respectively, became naturally preferred over continued size increase of the core (Figure 6a). Other accounts similarly documented the preparation of Ag@Fe_3_O_4_ CNHSs with a thick Fe_3_O_4_ layer upon solvothermal treatment of AgNO_3_ in the presence of Fe(NO_3_)_3_ and of ethylene glycol or sodium citrate + urea as reducing agents at 120–160 °C: in these circumstances, Fe_3_O_4_ was deposited upon reduction of Fe(NO_3_)_3_ onto the Ag nanocrystals that had been generated in the early reaction stages [200,201]. More recently, a one-pot, single-step templateless synthesis of micrometer-long magnetoplasmonic Au@Fe_x_O_y_ nanowires was achieved upon solvothermal treatment of HAuCl_4_ and FeCl_3_ (or Fe(NO_3_)_3_) precursors, and sodium acetate in a polyol mixture [202]. Following an analogous reaction pattern, different rates of pyrolysis of the relevant precursors were conveniently manipulated to synthesize Cr@γ-Fe_2_O_3_ and Co@γ-Fe_2_O_3_ [203], and FePt@Fe_3_O_4_ [204] core@shell CNHSs (Figure 6b,c). More recently, Au@CeO_2_ CNHSs, featured by tailored urchin-to-star shaped Au@CeO_2_ shell with a tunable degree of compactness, were derived by an aqueous route based on refluxing an aqueous solution of HAuCl_4_ and Ce(NO_3_)_3_ precursors in the presence of sodium citrate as reductant [205].

Three-component Ni@Au@ZnO CNHSs were fabricated by a simple one-pot two-step route [206]. Initially, asymmetric Ni@Au core@shell CHNSs were generated upon simultaneous reduction of HAuCl_4_(CO)_8_ and Ni(acac)_2_ by OLAM in benzyl ether (BE) at 200 °C. Subsequently, zinc stearate/OLAM/BE solution was introduced into the reaction medium and heated to 290 °C. Upon prolonged heating, a ZnO shell composed of multiple petal-like sections is deposited on the Ni@Au seeds, thus leading to Ni@Au@ZnO heterostructures with flower-like shape profiles. These CNHSs were found to be exploitable as magnetically recoverable, photocatalytic platforms (Figure 6d–f). More recently, a similar scheme was implemented to synthesize AuCu_3_@ZnO CHNSs carrying a polycrystalline ZnO shell with urchin-like to flower-like morphology [207]. Finally, an unusual prototype of chain-like magnetic Ni/Ni_3_C core@shell heteroarchitecture was obtained upon reduction of NiCl_2_ precursor in hot ethylene glycol at its boiling point, followed by partial carbonization of the Ni core with carbonaceous species derived from Ni-catalysed decomposition of some of the organic components in the reaction environment [208].

### 3.6. Thermally Induced Atomic Diffusion and Crystal-Phase Segregation

In certain circumstances, solid-state diffusion of atomic species across a pre-existing crystal lattice, followed by thermally driven phase segregation, may lead to the formation of heterostructures otherwise hardly achievable by conventional seeding techniques [209,210,211] (Scheme 2g). Leveraging on these mechanisms, for example, it was possible to synthesize electrocatalytically active Au@MnO core@shell CNHSs that could not be accessed by direct heterogeneous growth of MnO on Au [210]. In the reported account, Au_x_Mn_y_ seeds were first prepared upon reduction of manganese acetylacetonate with KBEt_3_H n in ODE/OLAM mixtures containing Au nanocrystal seeds at 240 °C, during which fast Mn diffusion into the Au led to the Au_x_Mn_y_ alloy nanocrystals. The Au_x_Mn_y_ were then subjected to oxidative annealing at 170° in distinct steps, which resulted in Mn extrusion and segregation toward the surface and its conversion to MnO upon reaction with O_2_. Ultimately, either Au@MnO core@shell CNHSs or multidomain Au-MnO CNHSs were formed, depending on the composition of the Au_x_Mn_y_ seeds [210]. According to another report, preformed Au-Ni heterodimer CNHSs, heated in the presence of tri-*n*-phenylphosphine as both stabilizers (for Au) and as a P reservoir at 230–270 °C, transformed into corresponding Au@Ni_12_P_5_ core@shell CNHSs [211]. The underlying growth sequence in the solution encompassed selective phosphorylation of Ni atoms to yield a Ni_12_P phase, crystallization and reconfiguration of the latter into a uniform-thick shell around the Au section of the starting Au-Ni heterodimer seeds. The presence of tri-*n*-phenylphosphine surfactant in the system was drawn to be essential to stabilizing the Au against the formation of an alloy with Ni [211].

## 4. Non-Core@Shell Heteromeric Architectures

A broad subfamily of CNHSs encompasses distinguished non-core@shell nanoarchitectures that feature a controlled (most frequently, nonsymmetric) spatial distribution of their atomic constituents and crystal phases. These CNHSs are represented by heteromeric constructs in which geometry- and phase-controlled domains of different materials are assembled through heterojunctions of small extension, such that a surface fraction of each component is also exposed to the external environment. In a sense, heteromeric CNHs may be considered as the inorganic equivalents of organic molecules provided with multiple functional groups [33]. In addition to gathering the properties of the component materials and enabling electronic coupling between them, heteromeric CNHSs can potentially allow regiospecific anchoring of molecular moieties to the available surfaces, thus offering surface platforms with spatially differential functionalization [5,22,23,24,25,27,28,29,32,33].

In seeded-growth synthesis, the evolution of a CNHS architecture can, in principle, be driven to change from a core@shell to a non-core@shell growth mode, depending on the conditions. Under thermodynamic control, the ultimate topology is decided by the global surface-interface energy change (see Equation (1)) that accompanies the heterogeneous deposition events and/or any other types of heterostructuring processes (e.g., phase segregation). For example, materials that can hardly alloy or intermix into solid solutions, and/or that are structurally mismatched may evolve into heteromeric architectures as far as this pathway permits minimization of the emerged interface strain at a comparatively lower price of augmented surface energy (arising from the multiple surfaces in contact with the liquid solution).

Equally, relatively smaller bonding heterojunctions may be easily attained between preformed CNCs if the formation of a heteroarchitecture is associated with an appreciable reduction in the global surface energy of the system, compared to that of a corresponding physical mixture of CNCs (which is likely to be the case when the starting CNCs these bear organic stabilizers weakly bound to the surface. Other conditions that may favour the formation of non-core@shell CNHSs may set in when growth is seeded with CNCs, of which only selected surface sites are physically accessible or chemically reactive. The capability of such CNCs to act as effective heterogeneous substrates may be governed by matching degree that may be met between the concerned lattices at the possible heterointerfaces, or by kinetic restrictions on the deposition process itself [5,22,23,24,25,27,28,29,32,33].

### 4.1. Heteromers Based on Nearly Isotropic-Shaped Material Domains

Several prototypes of heteromeric CNHSs grouping distinct size- and shape-controlled materials modules have been engineered. Such CNHSs hold significant potential for demanding technologies where multifunctionality and multitasking capabilities are desired (e.g., for the realization sensors, for the set-up of imaging techniques, and the performance of therapeutic actions in biomedicine, etc.) [22,23,24,25]. It has been considered that the distinct material sections of a heteromeric CNHS may serve as suitable grounds for the site-differential anchoring of biologically relevant molecules (e.g., DNA segments, short peptide chains, etc.), by exploiting the affinity of appropriate functional groups toward the available surfaces [22,23,24,25,212,213]. Moreover, while a metal or semiconductor domain in a CNHS can permit its identification optically, a magnetic module can be exploited for supplemental purposes, such as for MRI imaging and magnetic sorting [22,23,24,25,212,213,214,215,216,217,218,219,220]. Interestingly, in a CNHS, synergistic interactions setting through the heterointerfaces between the component materials can result in significantly altered magnetic [38,101,102,220,221,222,223,224,225], optical [226,227,228], transport [221], magneto-optical [39,40,41,42,43,44,229], and (electro)catalytic [230,231,232,233,234,235,236,237,238,239] properties, as well as energy-storing capabilities [240]. Such wealth of chemical-physical behaviour has both fundamental and practical relevance to the applicative horizons of suitably engineered CNHSs.

**Figure 7:**
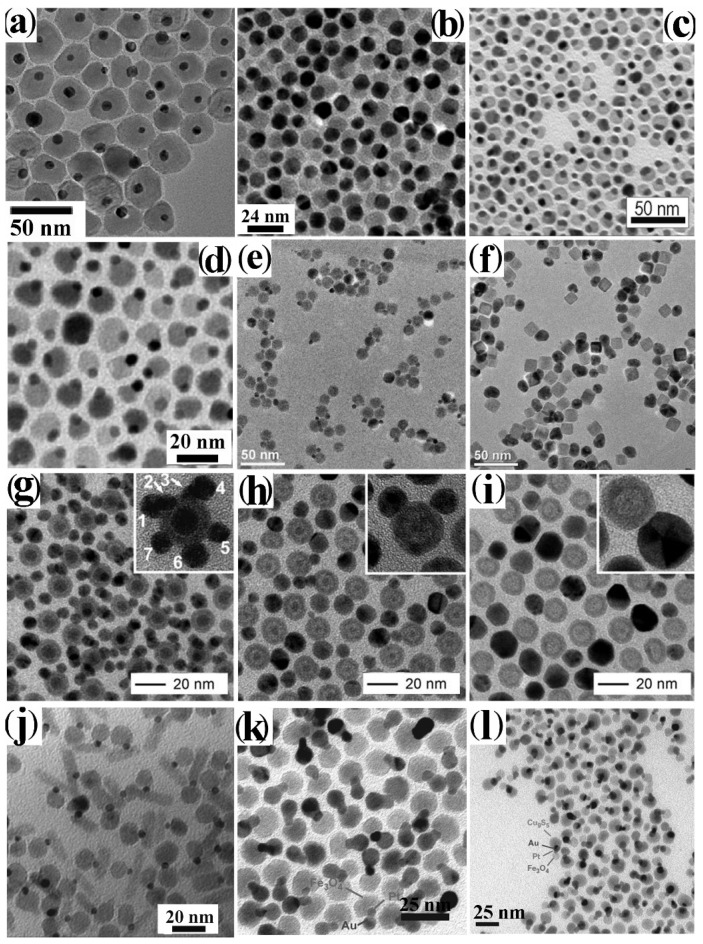
Gallery of representative heteromeric CNHSs obtained by direct heterogeneous nucleation-growth over preformed seeds (cf. Scheme 3a). The panel show TEM images of: (**a**) peanut-shaped Au–MnO hetero-dimer CNHSs (adapted with permission from Ref. [218], Copyright 2008, American Chemical Society); (**b**) dumbbell-like Au–Fe_3_O_4_ hetero-dimer CNHSs (reproduced from Ref. [241] with permission, copyright American Chemical Society); (**c**) Peanut-shaped FePt–In_2_O_3_ hetero-dimer CNHSs with cubic-shaped FePt domains (reproduced with permission from Ref. [250], Copyright 2011, American Chemical Society); (**d**) Peanut-shaped Pt–Fe_3_O_4_ hetero-dimer CNHSs (adapted with permission from Ref. [234], Copyright 2010, American Chemical Society); (**e**,**f**) asymmetric Fe_3_O_4_–Ag hetero-dimer CNHSs (reproduced with permission from Ref. [217], Copyright 2008, Wiley-VCH Verlag GmbH & Co. KGaA); (**g**–**i**) conversion of Fe@Fe_x_O_y_–Ag heteromer CNHSs to lower-order hollow-Fe@Fe_x_O_y_–Ag heteromer CNHSs to hollow-Fe_x_O_y_–Ag hetero-dimer CNHSs via ripening (reproduced with permission from Ref. [271], Copyright 2011, Wiley-VCH Verlag GmbH & Co. KGaA); (**j**) Au–Fe_3_O_4_–PbS hetero-trimer CNHSs obtained upon growing a rod-shaped PbS section out of the Au domain of Au–Fe_3_O_4_ hetero-dimer seeds (reproduced with permission from Ref. [99], Copyright 2006, American Chemical Society). (**k**,**l**) Ag–Pt–Fe_3_O_4_ and Cu_9_S_5_–Pt–Fe_3_O_4_ hetero-trimer CNHSs obtained by regioselective overgrowth of Au or Cu_9_S_5_ on the Pt domain of Pt–Fe_3_O_4_ hetero-dimer seeds (reproduced with permission from Ref. [100], Copyright 2012, Nature Publishing Group).

**Figure 8:**
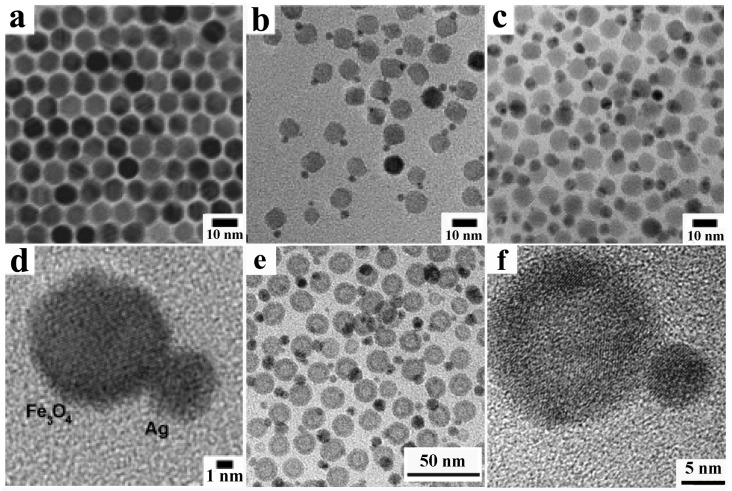
Gallery of representative heteromeric CNHSs produced at liquid/liquid interfaces (cf. Scheme 3b). The panels report variable-magnification TEM images of: (**a**) the starting Fe_3_O_4_ seeds and (**b**–**d**) Fe_3_O_4_–Ag hetero-dimer CNHSs thereof with different Ag domain sizes (reproduced with permission from Ref. [295], Copyright 2005, American Chemical Society); (**e,f**) Fe_3_O_4_–Ag hetero-dimer CNHSs with hollow Fe_3_O_4_ domain (adapted with permission from Ref. [296], Copyright 2010, American Chemical Society).

**Figure 9:**
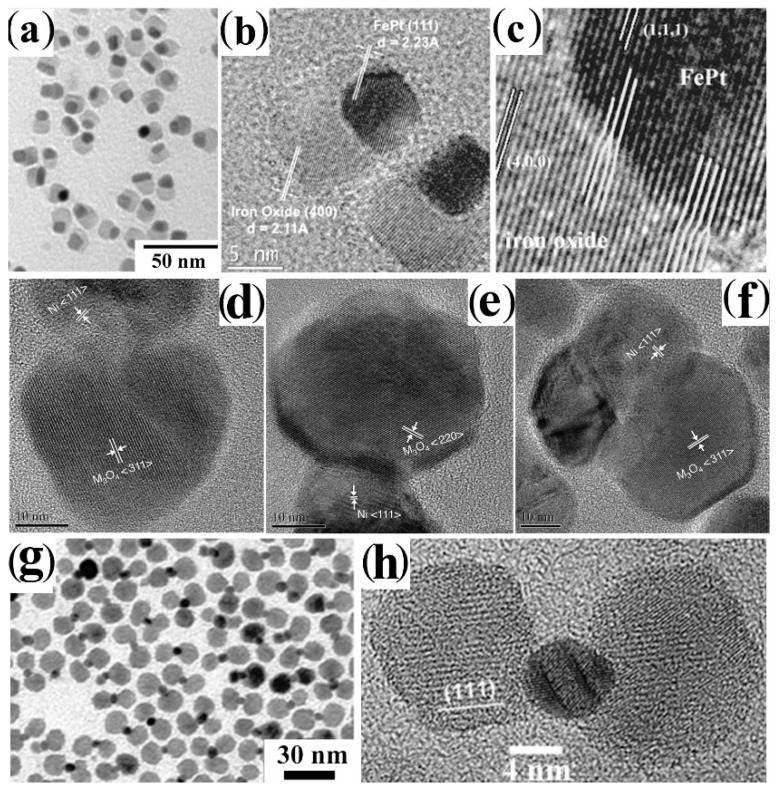
Gallery of representative heteromeric CNHSs formed by mechanisms that do not involve direct heterogeneous nucleation-growth over preformed seeds. (**a**) Low-magnification TEM and (**b**,**c**) HRTEM images of FePt–Fe_3_O_4_ hetero-dimer CNHSs formed upon self-controlled nucleation-growth (cf. Scheme 3c) (reproduced with permission from Ref. [297], Copyright 2008, American Chemical Society). (**d**–**f**) HRTEM images of FeNi-Me_x_O_y_ hetero-dimer CNHSs, derived by solid-state diffusion/phase segregation (cf. Scheme 3d) (reproduced with permission from Ref. [299], Copyright 2015, Royal Society of Chemistry). (**g**,**h**) TEM and HRTEM images of Fe_3_O_4_–Au–Fe_3_O_4_ hetero-trimer CNHSs with a Au domain connecting two Fe_3_O_4_ domains, derived from induced welding of preformed Au–Fe_3_O_4_ hetero-dimer seeds (cf. Scheme 3e) (reproduced with permission from Ref. [99], Copyright 2006, American Chemical Society).

**Scheme 3:**
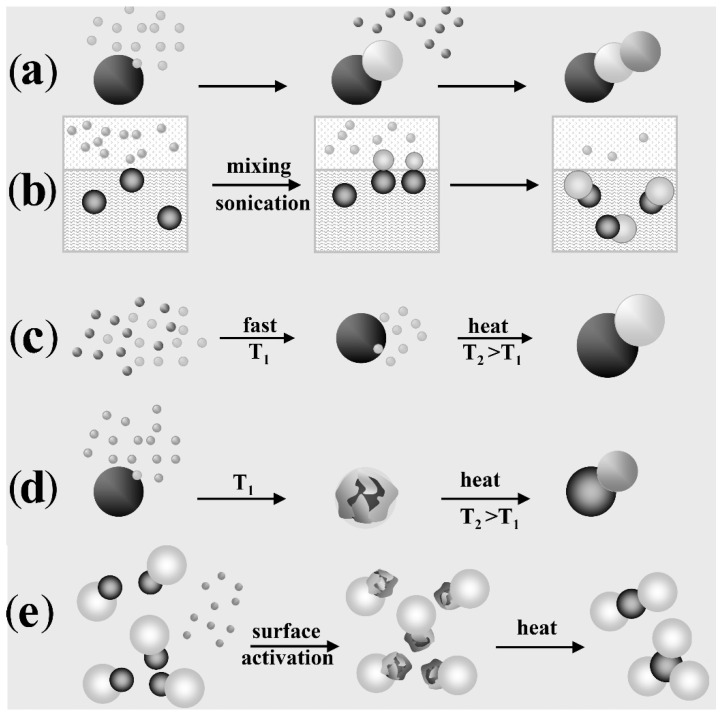
Sketch of the possible mechanisms that may lead to heteromeric CNHSs: (**a**) direct heterogeneous nucleation; (**b**) reactions at liquid/liquid interfaces; (**c**) self-regulated nucleation and growth; (**d**) solid-state atomic diffusion and phase segregation; (**e**) induced attachment of preformed nanocrystal heterodimers (adapted with permission from Ref. [27], Copyright 2008, Wiley-VCH Verlag GmbH & Co. KGaA).

Numerous CNHSs with hetero-dimer and hetero-oligomer arrangements, where groups of two or more isotropic-shaped (i.e., nearly spherical, or regular-polyhedral) modules are permanently assembled together, have been constructed by means of variants of the basic seeded-growth approach. A convenient criterion on the basis of which the various synthetic approaches may be categorized highlights the mechanism that accounts for heterostructure formation, as sketched in Scheme 3: (a) direct heterogeneous nucleation-growth in the solution phase (in some cases, accompanied by de-wetting); (b) heterogeneous nucleation-growth at the interface between immiscible liquids; (c) self-regulated homogeneous and heterogeneous nucleation-growth; (d) solid-state atomic diffusion and phase segregation and (e) induced welding of preformed nanocrystals. Representative TEM images that demonstrate the degree of synthetic elaboration afforded by such routes are collected in the following Figure 7, Figure 8 and Figure 9.

#### 4.1.1. Direct Heterogeneous Nucleation-Growth (and De-Wetting)

Direct heterogeneous nucleation (Scheme 3a) is the most intensively exploited and effective mechanism by which CNHSs can be engineered as two- or three-module heteromers composed of disparate combinations of metal and metal-oxide materials. A general synthetic procedure involves the thermal decomposition of metalorganic compounds or metal carboxylate complexes as the precursors in the presence of pre-synthesized metal, metal alloyFe_3_O_4_ or FePt seeds in a liquid environment composed of a solvent (typically, ODE or phenyl ether) loaded with selected surfactants (usually, OLAC, OLAM and/or TOP) at 200–300 °C. Such a scheme has enabled access to hetero-dimer CNHSs with epitaxially bound domains with close-to-spherical and/or cubic shape, such as of Au–Fe_3_O_4_, AuAg–Fe_3_O_4_, PtPd–Fe_3_O_4_, AuPd–Fe_3_O_4_, AuPt–Fe_3_O_4_, AuCu–Fe_3_O_4_, Pt–Fe_3_O_4_, Ni–Fe_3_O_4_, Cu–Fe_3_O_4_, Ru–Fe_3_O_4_, Au–CoO, AuAg–CoO, Pt–CoO, Au–MnO, Ag–MnO, FePt–CoFe_2_O_4_, FePt–In_2_O_3_, FePt–Fe_3_O_4_, FePt–MgO, Au–In:CdO, FePt–In:CdO, Pt–In:CdO, Pd–In:CdO, Cu–CeO_2_, Cu–ZrO_2_ and Cu–ZnO [99,218,219,224,230,231,232,233,234,237,238,239,241,242,243,244,245,246,247,248,249,250,251,252,253,254,255,256,257,258,259,260,261,262,263,264,265,266,267,268,269,270,271,272,273,274,275,276,277,278,279,280,281,282,283,284,285,286,287]. Synthesis under solvothermal conditions in polar media has also been investigated [249]. Depending on the geometric parameters of their component modules, these heterostructures could feature shape profiles spanning from peanut-, dumbbell- to brick- or flower-like (Figure 7a–d). In the vast majority of cases, the preference for the formation of a heterostructure made of spatially segregated discrete domains, over a core@shell configuration, has been assumed to be driven by the difference in a lattice structure and/or parameters between the component materials, which in fact imposes minimization of the contact area at the relevant heterojunctions to alleviate any associated misfit strain.

The formation mechanism of hetero-dimer CNHSs was carefully studied for the prototypical Au–Fe_3_O_4_ combination [241,245]. The solvent was concluded to play a critical role in regulating the possible sites for Fe_3_O_4_ nucleation over the Au seeds. The dumbbell-like configuration obtained in syntheses carried out in nonpolar media was believed to correlate with the induction of charge polarization across the seeds because of electron density enrichment at those locations of the Au seeds onto which Fe_3_O_4_ had firstly nucleated. Corresponding depletion of electron concentration at spatially opposed regions of the seeds was thus considered to inhibit the reiteration of heterogeneous nucleation thereon. Conversely, when growth was accomplished in a polar electron-donating solvent, any electron deficiency at the Au surface could be screened by the contact liquid. This condition renders the surface of the seed surface suitable to accommodate multiple nucleation events, thus allowing the growth of several “petals” or even a uniform coverage of Fe_3_O_4_ [234,241,242,243,244,245]. Recently, the formation mechanism of AuPt-Fe_3_O_4_ hetero-dimer CNHSs seeded with AuPt CNCs has been unveiled [238]. The evolution of these heterostructures involved the early formation of AuPt@Fe_3_O_4_ core@shell CNHSs (at 190 °C), followed by de-wetting of the Fe_3_O_4_ shell at higher temperatures (280–320 °C) and its reshaping into a segregated spherical Fe_3_O_4_ domain located aside [238]. Ultimately, the AuPt (seed) core carried a thin discontinuous Fe_3_O_4_ layer on one of its hemispherical surface portions and a major Fe_3_O_4_ domain on the diametrically opposite region. An analogous mechanism based on the de-wetting of a metastable Fe_3_O_4_ shell attained on the metal seeds in the early reaction stages may be expected to underlie the formation of other coinage-metal-Fe_3_O_4_ hetero-dimer CNHSs. This hypothesis was corroborated by the outcome of dedicated “tug-of-war” etching-destabilization experiments aimed at assessing the chemical reactivity of metal-Fe_3_O_4_ hetero-dimer architectures [235,238,262]. In these tests, Au could be selectively removed from Me-Fe_3_O_4_ (Me = Au, AuPt) hetero-dimers with peanut- to dumbbell-like profiles by with I_2_-driven leaching at ambient temperature. As a result, hetero-dimers with peanut- to dumbbell-like profiles were transformed into two types of nanostructure products: (i) nearly spherical all-Fe_3_O_4_ CNCs with a small concave region that corresponded to the space portion that was previously occupied by the nested Au domain of the starting hetero-dimer parents; (ii) peanut- and dumbbell-like solid/hollow all-Fe_3_O_4_ homo-dimer CNCs, where the hollow compartment enclosed a tiny Pt nanocrystal as the residue of the etching of the Au or AuPt domain of the parent hetero-dimers) [235,238,256,257,258,262]. The obtainment of such unusual Fe_3_O_4_ nanostructures with either engraved surfaces or with cavities upon selective metal leaching demonstrated that in a population fraction of the parent AuPt-Fe_3_O_4_ and Au-Fe_3_O_4_ CNHSs, the AuPt and the Au (hemi)domains, which had originally been assumed to be only partially nested into Fe_3_O_4_ section, could either expose the vast majority of their ‘’free’’ surface directly to the liquid medium, or have it protected by thin, either porous or discontinuous (thus, permeable) layer of Fe_3_O_4_. These observations are perfectly congruent with the participation of a de-wetting event in the early formation stages of the CNHSs, as described above. Other types of solid/hollow hetero-dimer CNCs were derived by performing chemical reactions (for example, sulfidation or etching and transmetalation) on either section of metal/metal-oxide hetero-dimer CNHSs [260,261].

Other accounts on the formation of metal/metal-oxide CNHSs documented that a transition from dumbbell- to flower-like configuration could be promoted at relatively higher temperatures and/or precursor-to-seed ratios [99,100,217,218,219,245,249,263]. Additionally, in several circumstances it emerged that the organic stabilizers acting in the reaction mixture (which could either pre-exist on the pristine seeds or be intentionally introduced), intervened decisively in determining which heterostructure configurations (hetero-dimer or core@shell) could be ultimately preferred. These facts suggested that the kinetics underlying the heterogeneous nucleation-growth process, affected by ligands and surfactants, could also play important roles in governing topology selection [217,218,219,221,241,242,243,244,245,249,251,263].

In order to explain the defined FePt to In_2_O_3_ lattice orientations found in FePt-In_2_O_3_ hetero-dimer CNHSs seeded with FePt nanocrystals [218], the crystallographic relations that could set between two lattices were comparatively analysed within the framework of the Coincidence Site Lattice Theory (CSLT) [264,265,266,267]. The degree of correspondence between the FePt and In_2_O_3_ crystal structures and the frequency with which the respective lattice points could be coincident or matching at the possible heterointerfaces were studied to identify which couples of facets of the two materials could be best bonding. The relative FePt to In_2_O_3_ lattice orientations found in the hetero-dimers could be predicted on the basis of an empirical bonding-energy criterion: in contrast to the common assumption that In_2_O_3_ would deposit on those facets of the FePt seeds that guaranteed minimum lattice misfit, the epitaxial deposition of In_2_O_3_ was considered to be directed onto those FePt facets at which the primary atomic layer of the deposited In_2_O_3_ could form the strongest chemical bonds with the FePt substrate underneath [250].

An inverted reaction sequence was devised to synthesize other metal/metal-oxide heteromeric CNHSs. For example, Fe_3_O_4_–Ag heterodimers were synthesized by reacting the metal precursor (an Ag(I)–ligand complex) with a mild reducing agent (such as alkyl amines, alkyl diols, Ar/H_2_ atmosphere) in the presence of the preformed Fe_3_O_4_ CNCs at modest temperatures (<120 °C) [217,268,269,270,271,272] (Figure 7e,f). For the case of γ-Fe_2_O_3_–Cu hetero-dimers, which were prepared by a conceptually analogous strategy, post-synthesis air oxidation of the metal sections was proven to yield corresponding γ-Fe_2_O_3_–Cu_2_O heterostructures that would otherwise be difficult to prepare by direct heterogeneous deposition [273]. The latter approach inspired the synthesis of CNHSs based on combinations of other materials [274].

As for what regards their physical-chemical properties, heteromeric CNHSs were commonly found to behave differently from their individual nanocrystal counterparts. For example, the fluorescence of semiconductor oxides was depressed because of the reduced radiative electron-hole recombination probability associated with metal-promoted electron relocation. The plasmon resonances on the noble metal components were observed to exhibit appreciable frequency shift and/or intensity damping due to the neighbouring dielectric environment [99,100,126,256,257,258,263,268,275,276,277,278,279,280,281,282]. Also, relevant magnetic parameters deviated from those of the individual magnetic domains [216,248,256,257,258,270,283,284,285,286], indirectly denoting that interfacial interactions between nonhomologous materials established across CNHSs could affect their electronic properties and, consequently, their ultimate behaviour (e.g., catalytic and hyperthermal properties) [99,100,217,256,257,258,259,268,269,276,280,287].

The formation mechanisms of hetero-dimer CNHSs with deliberately tuneable domain sizes have been deciphered for many materials associations and found to share common aspects. In a broader physical-chemical perspective, the CNCs utilized as seeds may be regarded as heterogeneous catalysts for the chemical reaction selected for generating the targeted secondary material. The degree of reactivity of the seeds critically depends on their size, crystal structure, and geometry (hence, on facet distribution at the surface and local curvature). The relative seed to reactant proportions and temperature govern the size to which the newly implanted materials modules could ultimately develop by regulating the flux of reactive monomer approaching their surfaces [217,270,271,272,285,287].

On a thermodynamic basis, when local structural-geometric constraints on heterogeneous nucleation, such as excessive surface curvature, nonnegligible lattice mismatch and weak chemical bonding at the possible heterointerfaces, prevent secondary deposition events from taking place ubiquitously and at similar rates over a nanocrystal seed, then the formation of a uniform and seed-conformal shell cannot be achieved [5,8,9,10,11,28,29]. In other words, in circumstances under which the seeds are enclosed by structurally and chemically nonequivalent facets and/or expose particularly unstable surface features (edges, corners), such that deposition becomes preferred at selected sites where interfacial strain is minimized and reactivity is higher, heteromer arrangments become favored over core@shell configurations [217,280,285,286,288] (Scheme 1a).

The general arguments above explain why, starting from relatively small nanocrystal seeds, which expose multiple narrow facets and dense distribution of asperities (hence, high local curvature), hetero-dimer CNHSs are more likely to be obtained. On the other hand, bigger nanocrystal seeds, which generally offer equivalent sets of larger stabilized facets, can accommodate simultaneous deposition events at several locations, where nucleation is kinetically preferred and/or misfit strain is comparatively lower (Scheme 1b). In such cases, hetero-oligomer CNHSs can evolve with flower-like or urchin-like architectures, in which a central nanocrystal domain, corresponding to one of the starting seeds, is connected to multiple “petals” of a foreign material arranged around [115,249,265,266,271,272,285,286,287,288,289,290,291]. Strategies based on the selection of appropriate surfactants [235,248] or calibrated reducing conditions (e.g., in Ar/H_2_ atmosphere [286]) have been used to either exacerbate or mitigate differences in reactivity among the exposed facets, thus allowing control over the probability density of heterogeneous nucleation over the seeds.

In a detailed account of heterogeneous nucleation of Ag seeded with spherical Fe@Fe_x_O_y_ core@shell CNHSs in an organic mixture [271], high-order multiply Ag-decorated Fe@Fe_x_O_y_–Ag heteromer CNHSs were observed to transform to lower-order hollow-Fe@Fe_x_O_y_–Ag heteromer CNHSs, then, to hollow-Fe_x_O_y_–Ag hetero-dimer CNHSs (Figure 7g–i). Across this evolution, the mean dimensions of the Ag domains and the width of the Fe_x_O_y_ shell increased, while the Fe cores of the seeds contracted in size until disappearance. The distribution of the density and dimensions of the Ag domains over time was consistent with the standard LaMer nucleation and Ostwald ripening pictures. Concomitant to the Ag growth, the Fe@Fe_x_O_y_ sections of the seeds were observed to enter a Kirkendall diffusion regime upon gradual oxidation of the Fe cores presumably performed by O_2_, Ag^+^, and/or NO_3_^−^ residuals present in the reaction environment (cf. Scheme 2e). This process eventually resulted in the formation of capsule-like Fe_x_O_y_ domains with thick walls [271].

The ‘’total synthesis’’ paradigm that underlies the fabrication of complex organic molecules carrying multiple functional groups has inspired approaches for the elaboration of sophisticated CNHSs made of a high number of component domains. Heteromer nanoarchitectures have been constructed across programmed sequences of regioselective seeding steps over CNHS seeds (Scheme 3a) in which the targeted materials are generated by exploiting established chemical routes. For example, Fe_3_O_4_–Au–PbSe hetero-trimer CNHSs (Figure 7j) were engineered by inducing PbSe to nucleate selectively on the Au domains of Fe_3_O_4_–Au dumbbell-like hetero-dimer CNHSs upon the reaction of Pb- and Se-based complexes in hot surfactant mixtures [99,100,290]. The PbSe domains appeared to evolve to anisotropic rod-like shapes from the Au substrate through a solution-liquid-solid (SLS) growth pathway [5]. After growth completion, the PbSe sections detached from the seeds and were released as free-standing nanorods into the liquid medium [290] (Figure 7j).

In a similar way, hetero-trimer CNHSs with aligned domain arrangement and Fe_3_O_4_–Pt–Au, Fe_3_O_4_–Pt–Ag Fe_3_O_4_–Pt–Ni, Fe_3_O_4_–Pt–Pd, Fe_3_O_4_–Pt–PbS or Fe_3_O_4_–Pt–Cu_2_S composition (Figure 7k,l) were constructed by adding a third material to Fe_3_O_4_–Pt hetero-dimer CNHSs. The synthesis yielded only one hetero-trimer isomer, where the metal or metal-sulphide domain was selectively attached to the Pt section of the starting Fe_3_O_4_–Pt seeds [100]. Such result was interpreted as a nanocrystal-related analogue of regiospecificity in organic synthesis, in a context where, among the possible molecular isomer products (characterized by distinct organization of their functional moieties) that may potentially be obtained, only one is eventually obtained. Appropriate control experiments suggested that chemoselectivity of the deposition reaction over the Fe_3_O_4_–Pt seeds could be driven by electron enrichment across the Pt domain due to charge transfer from its Fe_3_O_4_ neighbour. Further microscopic insight into the formation of Ag–Pt–Fe_3_O_4_ heterotrimer CNHSs [292] unveiled that Ag initially nucleated onto both the Pt and Fe_3_O_4_ domains of Fe_3_O_4_–Pt seeds, then the Ag atoms tended to diffuse over the surface and coalesce into a single Ag domain attached aside the Pt section, thereby yielding the final Fe_3_O_4_–Pt–Ag heterotrimer isomer. The size of the Ag domain in the Fe_3_O_4_–Pt–Ag hetero-trimer CNHSs appeared to correlate with the extension of the surface exposed by Fe_3_O_4_–Pt seeds. This fact could reflect the occurrence of a process of Ag congregation proceeding through a surface-mediated transfer. Additionally, it was found that tiny Fe_3_O_4_ islands, which pre-existed on the Pt section of the Fe_3_O_4_–Pt seeds, played a role in governing the shape of the Ag domain [292].

To synthesize other hetero-trimer CNHS isomers, a technique based on the use of a solid-state protecting group was applied [293]. The proposed methodology involved a preliminary coating of the Pt domains of the starting Fe_3_O_4_–Pt hetero-dimer seeds with a thin amorphous Fe_x_O_y_ layer that served as a solid-state protecting group to isolate the Pt moiety. After this seed modification step, a third Ag or Au domain could be re-directed to nucleate on the Fe_3_O_4_ domain, a location where the metal deposition was otherwise disfavoured. This strategy thus permitted achieving the distinct and otherwise inaccessible Ag–Fe_3_O_4_–Pt and Au–Fe_3_O_4_–Pt hetero-trimer CNHS isomers, respectively [293].

Finally, it deserves mentioning that, in some cases, heterogeneous nucleation events have been observed to interfere and/or compete with transformative pathways, e.g., galvanic replacement, ion-exchange reactions, and Kirkendall diffusion. However, in such circumstances, the as-derived nanoheterostructures featured an irregular spatial distribution of their chemical composition or entailed a hollow region in one of their constituent material modules [192,276,277,294].

#### 4.1.2. Heterogeneous Nucleation-Growth at the Interface between Immiscible Liquids

An implemented technique to prepare hetero-dimers made of one magnetic and one coinage-metal domain built upon accomplishing seeded growth within the nanoscale-confined environment of a liquid/liquid heterointerface under mild conditions [295,296]. Prototypes of CNHSs achieved by this bi-phasic scheme are shown in Figure 8. In the devised procedure (Scheme 3b), an aqueous metal-salt solution was combined with an immiscible organic solvent (e.g., dichlorobenzene, dichloromethane, hexane, DOE) that contained preformed surfactant-protected γ-Fe_2_O_3_/Fe_3_O_4_ or FePt CNC seeds under inert atmosphere. Ultrasonic irradiation was then applied to promote formation of an oil-in-water emulsion, where the continuous aqueous phase contained “colloidosomes”, organic micron-sized droplets stabilized by the hydrophobic seeds adsorbed at the organic/water heterointerfaces [295]. During emulsification, the CNC seeds exposed catalytic-active sites onto which the Ag^+^ or AuCl_4_^─^ ions could be reduced to Ag or Au, respectively, by the ultrasonically produced radicals. Because the seeds were only party in contact with the aqueous phase, metal nucleation and growth stayed spatially confined to a small surface portion and proceeded auto-catalytically. As a result, a single metal domain was grown on each seed (Figure 8a–d). The approach was further elaborated and extended to synthesize solid Ag–hollow γ-Fe_2_O_3_ hetero-dimer CNHSs, which were seeded with hollow γ-Fe_2_O_3_ CNCs [257] (Figure 8e,f). All hetero-dimer CNHSs synthesized by the colloidosome–based route served as dual-surface platforms onto which a programmed surface distribution of biomolecules could be implanted to make them exploitable for various biomedical applications [212,213,296].

#### 4.1.3. Self-Regulated Homogeneous and Heterogeneous Nucleation-Growth

A few studies have documented the possibility of synthesizing hetero-dimer CNHSs by one-pot approaches that do not require the preliminary preparation and purification of appropriate nanocrystals to be used as the starting seeds. In these cases, all material precursors required to build up the heterostructures are introduced all at once into the same mixture. Prior knowledge of the relative reactivity order of the reactants in the synthesis environment that is intended to be realized is crucial to the success of the whole procedure. Indeed, under carefully set kinetic conditions, the nucleation-growth processes that may lead to the formation of each target materials may take place temporally separated from each another, to the point of allowing sequential formation of the different modules that will compose the final heterostructure (in a sense, the homogeneous and heterogeneous nucleation-growth events “self-govern”). Examples of CNHSs delivered by such a mechanism are displayed in the top part of Figure 9.

One significant demonstration is offered by the construction of FePt–Fe_x_O_y_ hetero-dimer CNHSs with adjustable geometric parameters (Figure 9a–c). These were achieved upon combining platinum acetylacetonate and Fe(CO)_5_ precursors in a hot OLAM/OLAC/ODE environment [297] (Scheme 3c). The two domains were generated consecutively across distinct periods. In the beginning, homogeneous nucleation-growth of FePt CNCs occurred at T ≤ 200 °C; then, a thin polycrystalline Fe_x_O_y_ coating grew on the pre-existing FePt seeds at T ≈ 295 °C; in response to the intensification of mismatch strain, the unstable Fe_x_O_y_ shell de-wetted out and reshaped into a discrete spinel-phase Fe_x_O_y_ hemispherical domain adjoined to FePt. As each construction step was induced and promoted in a specific range of temperatures, adjustment of the heating ramp allowed the two sections of the CNHSs to evolve at distant times during the heating period. These CNHSs were found to be biocompatible [298] Because of the exchange-spring coupling setting through the heterointerface between FePt and Fe_x_O_y_, the CNHSs manifested not only the typical magnetic response of a single magnetic phase, but also enhanced MRI contrast-agent properties, relative to those of the single constituents [297].

#### 4.1.4. Solid-State Diffusion and Phase Segregation

Combined atomic diffusion and phase-segregation pathways, analogous to those intervening in the formation of some core@shell CNHSs, have been assessed to promote the evolution of heteromer-type CNHSs. Several prototypes of CNHSs and nanocomposite architectures were obtained by leveraging on these mechanisms. The common synthetic scheme consisted in thermally or chemically inducing extrusion of selective atomic species from preformed CNHSs capable to react and form a separate, yet still adjoined phase (Scheme 3d). Examples of CNHSs derived by such route are shown in middle part of Figure 9.

One significant account documented the formation of NiFe-Me_x_O_y_ (Me = Ni, Fe) hetero-dimer CNHSs upon the reaction of α-Fe CNCs with nickel acetylacetonate in an ODE-diluted mixture of OLAM and hexadecylamine at 180 °C [299]. Under the specified synthesis conditions, the metal seeds underwent partial galvanic replacement and alloying with Ni, followed by rapid partial oxidation. As a result, the final heterostructures comprised joint polycrystalline domains of NiFe and of Ni/Fe ferrites (Figure 9d–f).

Interesting prototypes of hard nanomagnets made of exchange-coupled *fct*–FePt/α-Fe, *L1*_0_–FePt/α-Fe, and *L1*_0_–FePd/α-Fe phases were all obtained by a strategy that relied on inducing extrusion of selective atomic species from preformed CNHSs to form a separate reactive phase. In the reported case studies, preformed γ-Fe_2_O_3_–FePd, γ-Fe_2_O_3_–FePt or γ-Fe_2_O_3_–Pd heteromeric CNHSs were subjected to thermal annealing in Ar/H_2_ atmosphere [252,254].

During this process, the original γ-Fe_2_O_3_ phases were reduced, leading to Fe atoms that diffused and coalesced into a segregated α-Fe phase. The nascent α-Fe was reactive enough to form bonding heterojunctions with the stable FePt and FePd alloy domains of the starting γ-Fe_2_O_3_–FePt and γ-Fe_2_O_3_–FePd substrates, while it could even alloy with the Pd domain of γ-Fe_2_O_3_–Pd seeds. Ultimately, nanocomposites made of interconnected α-Fe and *fct*–FePt, L1_0_–FePt and *L1*_0_–FePd grains were obtained in the respective cases [252,253,254]. On the line of these achievements, more recently, preformed metal and metal-oxide CNCs have been proposed to be exploitable as precursors for high-temperature solid-state synthesis of ternary metal oxides [300].

#### 4.1.5. Induced Welding between Preformed Nanocrystal Hetero-Dimers

A smart strategy with the potential to augment the degree of architectural complexity of nanoheterostructures conceives preformed CNHSs as the inorganic analogues of small molecules that are forced to react with each other through their functional group(s) to form bulkier molecules (Scheme 3e). Examples of CNHSs that were synthesized on application of this paradigm are shown in bottom part of Figure 9. Fe_3_O_4_–Au–Fe_3_O_4_ hetero-trimer CNHSs, composed of one Au core bridging two Fe_3_O_4_ sections (Figure 9g,h), were synthesized upon inducing the Au domains belonging to distinct Au–Fe_3_O_4_ hetero-dimer CNHSs to solder to each other, in the presence of molecular sulphur as the attachment promoter [99]. Due to its high binding strength to gold, S atoms were assumed to adsorb on the Au portions of the starting hetero-dimers and trigger detachment of the bound surfactants from the Au surfaces. This condition thus promoted fusion between Au domains upon collision. Thermodynamically, such evolution can be interpreted as being accompanied by a reduction in the overall surface energy of the system. Further elaboration of this reaction scheme permitted access to higher-order heteromer CNHSs with linear or intricate ramified topologies. For example, CNHSs with a Fe_3_O_4_–Pt–Au–Au–Pt–Fe_3_O_4_ profile were obtained upon sulphur-aided regioselective congregation of preformed Au–Pt–Fe_3_O_4_ hetero-trimer CNHSs, which were eventually driven to connect through their Au domains [100]. 

### 4.2. Nanoheterostructures Based on Anisotropic-Shaped Domains

CNHSs with more sophisticated topological profiles are based on nanoarchitectures that incorporate anisotropic material sections with planar, linear, or branched profiles, which feature peculiar shape-dependent physical-chemical properties [5,118]. Most suitable to construct CNHSs with anisotropic shape motifs are semiconductor compounds (metal chalcogenides, metal phosphides, and metal oxides) which crystallize in low-symmetry (e.g., hexagonal, tetragonal, orthorhombic) phases. Such materials have a strong propensity to develop preferentially along one crystallographic direction, most commonly along or orthogonal to the direction of their axis of higher symmetry. After homogeneous or heterogeneous nucleation, nanostructures of such low-symmetric phases can thus easily evolve into nanoplatelets, nanowires, nanorods, or polypods, especially when growth is assisted by a facet-selective surfactant adhesion mechanism [5,8,9,10,11,28,29,34,36,291]. In the case of metal materials, which crystallize in centrosymmetric phases, anisotropic growth can still be achieved as a consequence of the induction of structural deviations that interrupt the symmetry of isotropic embryos in the early development stages [301,302,303].

Shape anisotropy has dramatic implications on the seeding behavior of these CNCs whenever they are employed as starting substrates for the construction of a heterostructure. The facets that enclose the apexes, edges, and longitudinal walls are most commonly characterized by dissimilar atomic configurations, and/or topological irregularities arising from symmetry breaking during growth. Such locations thus show distinct chemical reactivity and/or structural stability. In rod-/wire-shaped CNCs, the absence of a plane of symmetry orthogonal to their principal lattice-development axis implies that the two basal facets at the terminations are crystallographically, hence chemically, inequivalent. Tapering of the apex profile may accentuate such differences [266,304,305,306,307,308,309,310,311,312,313]. Consistent with the mechanism of their anisotropic evolution, the longitudinal sidewalls and the apexes of rod-/wire- and polypod-tailored CNCs can manifest different propensities to accommodate foreign materials domains when they are utilized to seed their heterogeneous growth. Moreover, depending on the particular case, anisotropic CNCs, whose crystallographic structure deviates from that of their bulk reference and/or whose shape has reduced symmetry, can feature an intrinsic dipole moment or expose surface defects, which may affect their seeding behaviour [36,291,304,310,311,314].

Thermodynamic arguments suggest that deposition of any foreign materials on anisotropic-shaped seeds would be preferred at those sites that correspond to the lowest-energy configuration permitted by the surface-interface tension equilibrium at all formed heterointerfaces (Equations (2) and (3)) [5,8,9,10,11,32,36,291]. For example, selective heterogeneous nucleation can be thermodynamically favoured if it leads to the disappearance of unstable facets of the seeds (e.g., those located at the terminations of nanorods/nanowires) at the relatively lower cost of the misfit strain energy required for the formation of the new heterointerfaces. However, when nucleation and growth take place under kinetic control, and/or are complicated by occurrence of chemical or physical transformations (e.g., solid-state atom diffusion, ion exchange, crystal-phase conversion), it remains hard to decipher which mechanisms decide topology selection [5,8,9,10,11,27,32,36,291,306,307,308,309,315,316].

At the relevant heterojunctions, imperfect coincidence of lattice points leads to breaking of the crystal periodicity, giving rise to interfacial strain, which may, in turn, affect the electronic-band alignment [34,36] and electronic coupling between the connected domains [310,315,317,318,319]. The nominal misfit strain may be relaxed via diverse mechanisms, such as: (i) the formation of crystal defects (e.g., dislocations stacking faults) or local deformation of the near-heterointerface lattice (coherency strain), [300,320], both of which are processes allowed at the temperatures at which liquid-phase syntheses are normally conducted; and/or (ii) via graduation in the chemical composition across the heterojunctions, driven by atomic inter-diffusion across the adjoined materials [321,322]. Whether alloyed, graded or abrupt interfaces are formed will heavily depend on the specific growth conditions realized [300,310,315,316,317,318,319,320,321,322,323].

As found for their core@shell counterparts, in anisotropic CNHSs built of metals and metal-oxide materials, the electronic structure across the heterointerface may be deliberately controlled by designing configurations suitable to guarantee confinement or favor spatial separation of the excited electron-hole pairs [32,34,36,291]. The magnetic behavior may result in being severely altered, with respect to that of their isolated components, even when in the heterostructure magnetic materials are bound to non-magnetic ones [267,268,270,275,277,278]. CNHS nanoarchitectures have been demonstrated to hold great potential as functional elements for the engineering of devices for optoelectronics, photocatalysis [32,36,291], spintronics and magnetic recording [210,307,308,315,317,318,319].

**Figure 10:**
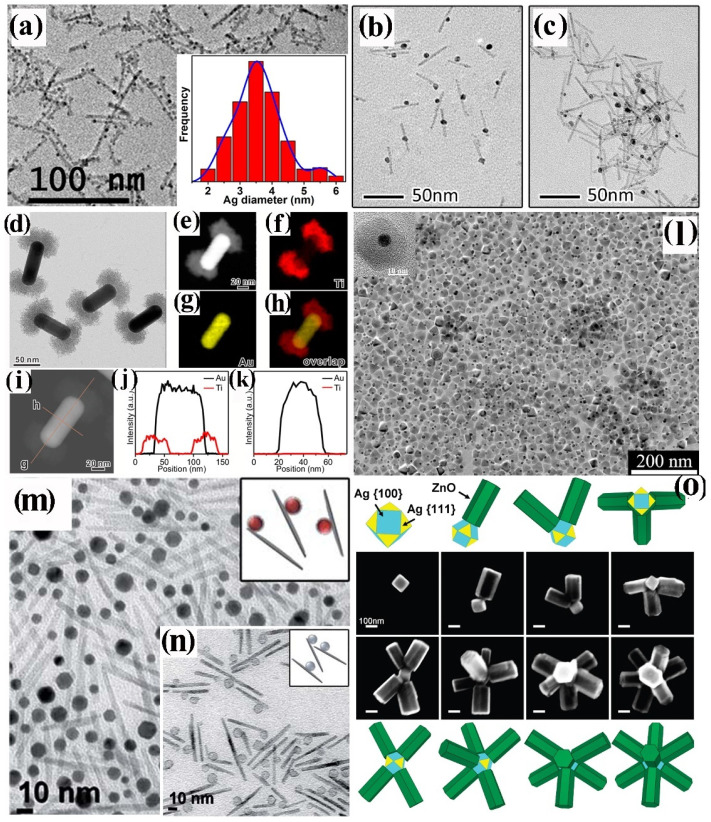
Gallery of representative two-component anisotropic CNHSs synthesized by site-selective heterogeneous deposition on preformed seeds with variable shapes (cf. Scheme 4). (**a**) TEM image of Ag-TiO_2_ CNHSs composed of multiply Ag–decorated TiO_2_ nanorods; with the Ag size histogram in the inset (reproduced with permission from Ref. [338], Copyright 2019, American Chemical Society); (**b**,**c**) TEM images of binary Ag–TiO_2_ CNHSs composed of asymmetrically Ag–decorated TiO_2_ nanorods, formed via a photocatalytically driven intraparticle ripening after different UV irradiation times (reproduced with permission from Ref. [340], Copyright 2013, American Chemical Society). (**d**) TEM image, (**e**–**h**) elemental EXD maps, (**i**) HAADF-STEM image and elemental EDX profiles (**j**,**k**) of nanodumbbell-like CNHSs individually composed of one Au nanorod equipped with extended TiO_2_ heads at the apexes (reproduced with permission from Ref. [341]; Copyright 2016, American Chemical Society). (**l**) TEM image (HRTEM image in the inset) of Au–ZnO CNHSs composed of a ZnO nanopyramid connecte to a single Au domain at its basal side (reproduced with permission from Ref. [342]; Copyright 2011, American Chemical Society). (**m**) TEM images of Au–Fe_x_O_y_ CNHSs made of asymmetrically Au@Fe_x_O_y_-functionalized Fe_x_O_y_ nanorods and (**n**) corresponding all-oxide nanostructures obtained upon Au leaching (reproduced with permission from [344], Copyright 2011, Royal Society of Chemistry). (**o**) Scanning Electron Microscopy (SEM) images of polypod-like CNHSs composed of multiple ZnO nanorod branches grown out of the (111) facets of Ag truncated-nanocube seeds; the corresponding geometrical models of the heterogeneous nanostructures are shown (the yellow and cyan planes represent the (111) and (100) facets of Ag, respectively; the green planes represent the facets of the ZnO nanorods) (reproduced with permission from [345], Copyright 2009, American Chemical Society).

**Figure 11:**
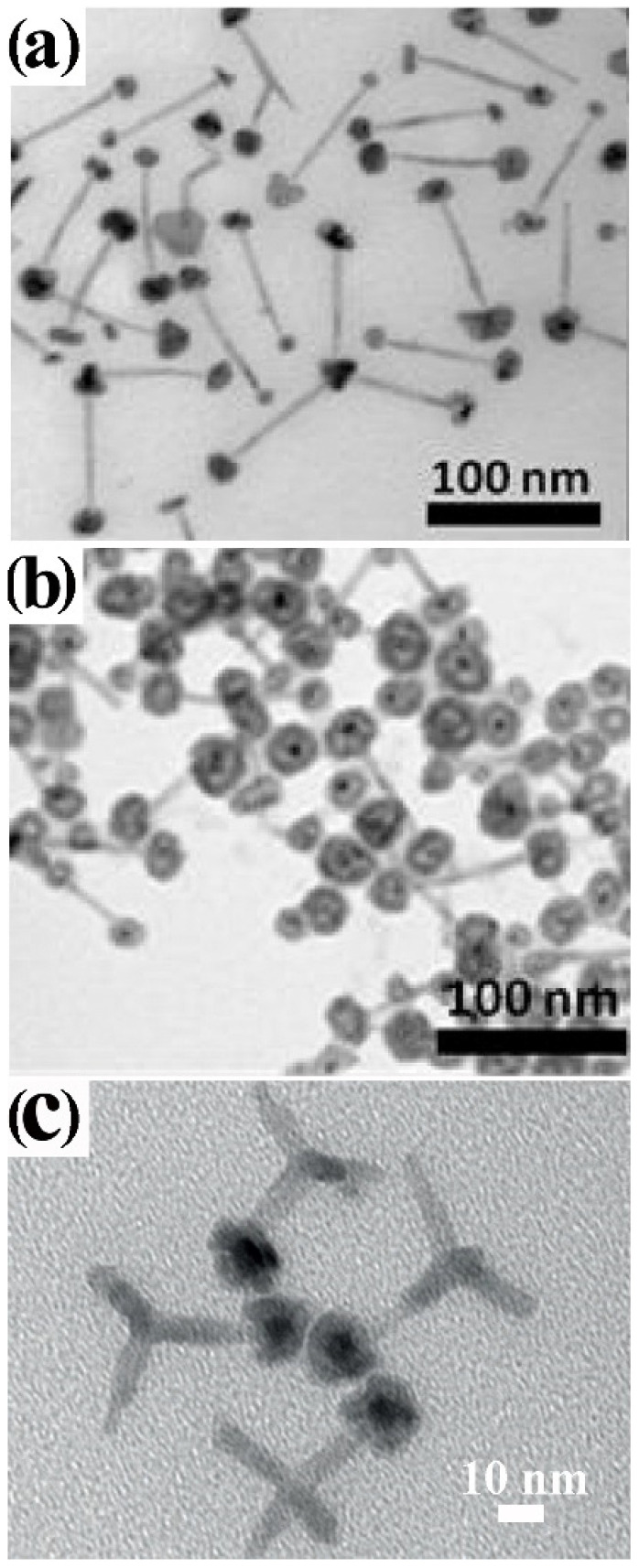
Representative anisotropic CNHSs synthesized by regioselective heterogeneous deposition on preformed rod-like and polypod-like seeds (cf. Scheme 4). (**a**) Nanodumbbel-like CNHSs composed of Pt@Co–tipped CdS nanorods, which were obtained overgrowing Co domains on the apexes of preformed Pt–tipped CdS nanorods and (**b**) nanodumbbel-like CNHSs made of Pt@Co_x_O_y_–tipped CdS nanorods with Pt@Co_x_Oy yolk@shell heads, which were prepared by oxidizing the Co domains of parent Pt@Co–tipped CdS nanorods (reproduced with permission from Ref. [346], Copyright 2012, American Chemical Society; (**c**) CdSe@CdS core@shell tetrapods asymmetrically decorated with a single Au@Co/Co_x_O_y_ core@shell head (reproduced with permission from Ref. [348], Copyright 2016, Wiley-VCH Verlag GmbH & Co. KGaA).

**Figure 12:**
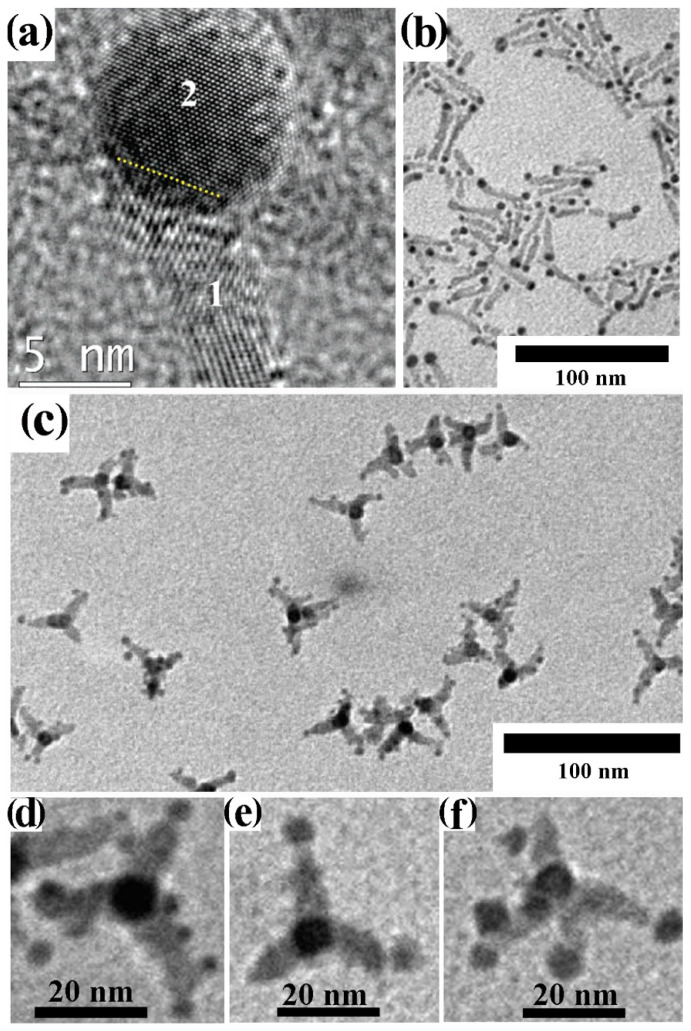
Collection of representative anisotropic CNHSs obtained by a surfactant-controlled selective heterogeneous nucleation-growth mechanism (cf. Scheme 4). The panel show: (**a**,**b**) HRTEM image (where “1” denotes the TiO_2_, while “2” denotes Co) and TEM image of CNHSs composed of Co-tipped anatase TiO_2_ nanorods (reproduced with permission from Ref. [315], Copyright 2007, American Chemical Society); (**c**–**f**) variable-magnification TEM images of heteromer CNHSs made of Co-decorated γ-Fe_2_O_3_ tetrapod-shaped nanocrystals (reproduced with permission from Ref. [318], Copyright 2009, American Chemical Society).

**Scheme 4:**
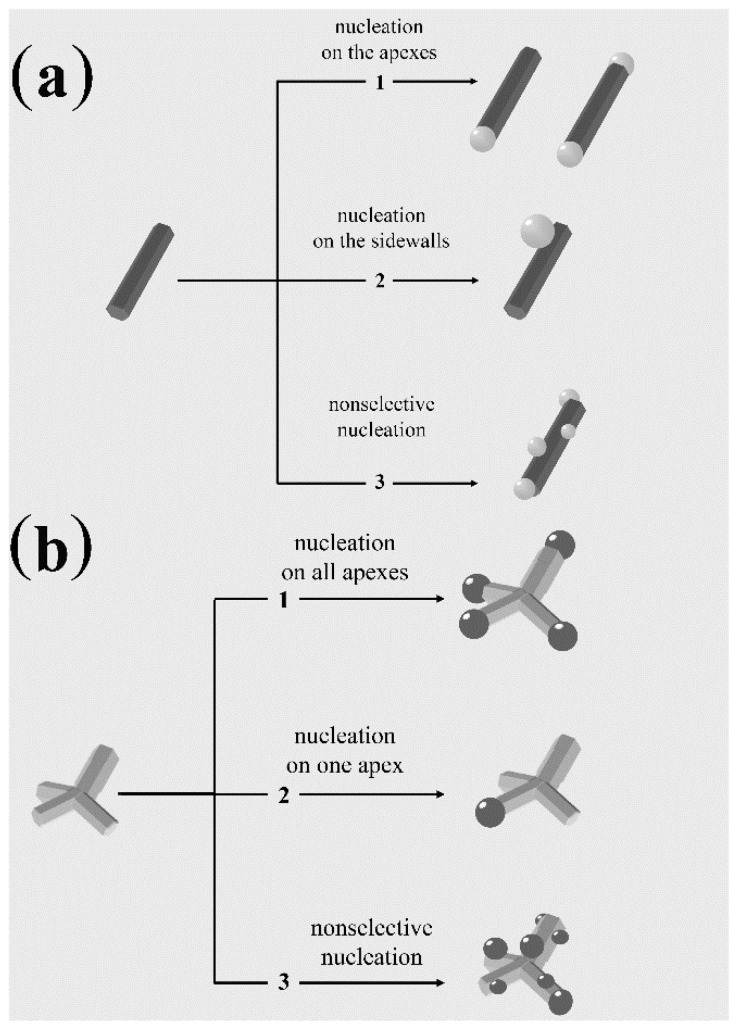
Sketch of the possible routes that may yield anisotropic CNHSs, starting from linear- and branch-shaped nanocrystal seeds: decoration of a rod-/wire-shaped seed (**a**) or of a tetrapod seed (**b**) with isotropic-shaped domains of a secondary material at specific sites (reprinted with permission from Ref. [26], Copyright 2007, Bentham Science Publisher).

The main mechanisms on which the construction of CNHSs based on anisotropic (e.g., linear, branched) material sections has leveraged are sketched in Scheme 4. They include: (i) regioselective heterogeneous nucleation-growth; (ii) surfactant-controlled facet-selective deposition. Examples demonstrating the degree of architectural sophistication achieved so far are collected in Figure 10, Figure 11 and Figure 12.

#### 4.2.1. Regioselective Heterogeneous Nucleation

Among the most controlled and accurately structured breeds of anisotropic CNHSs achieved by heterogeneous nucleation-growth are earlier-developed prototypes metal-semiconductor heterostructures, constructed by exploiting preformed metal-chalcogenide rod-shaped CNCs as starting platforms for the growth of near-isotropic domains of a metal [281,308,322,324,325,326,327,328] (Scheme 4a). For these systems, the secondary metal domains could be located at the nanorod seed apexes with a high degree of regioselectivity [325,326,327,328,329]. Less frequently, an opposite reaction scheme has been proposed, where CNC seeds of the target metal are utilized to promote heterogeneous nucleation and accommodate growth of anisotropic-shaped sections of a semiconductor compound [330]. Building upon on this synthetic success, the development of several prototypes of CNHSs based on metal and metal-oxides has been pursued, yet a comparatively lower degree of configurational control has generally been achieved [331,332,333,334,335,336,337,338,339,340,341,342,343,344,345,346,347,348]. Relevant cases are illustrated in Figure 10 and Figure 11.

A vast majority of protocols involved the exploitation of organic-uncapped oxide metal-nanorods as seeds, on which the positions and surface density of foreign metal domains installed upon heterogeneous nucleation could hardly be controlled, thus preventing synthetic switching between distinct isomers (Scheme 4a, paths 2–3). For example, CNHSs made of Ag-decorated ZnO nanorods were synthesized by a one-pot surfactant-free solvothermal method relying on basic hydrolysis of zinc acetate and reduction of silver acetate at 160 °C in ethanol [331]. Au-decorated ZnO nanorod CNHSs were synthesized by inducing the reduction of HAuCl_4_ over ZnO nanorods by means of a copper foil in the presence of CoCl_2_ as inhibitor of parasitic independent nucleation [332]. In both cases, the as-derived CNHSs exhibited enhanced photocatalytic performances under UV irradiation, with respect to those of the ZnO component alone. This behaviour stemmed from metal-promoted interfacial separation of the photoexcited carriers in the semiconductor. Interesting binary CNHSs made of Ag–decorated Zn_0.9_Co_0.1_O nanorods were produced by a one-pot solvothermal approach [333,334]. Compared to the starting paramagnetic Zn_0.9_Co_0.1_O nanorod seeds, the heterostructures exhibited ferromagnetic behaviour at room temperature.

A family of photocatalytically active TiO_2_–metal (metal = Ag, Pt, Ru, PtRu) CNHSs was synthesized by a one-pot scheme, according to which hydrolysis of TiCl_3_ generates unstable Ti(III)-oxide nanorods that converted to rutile TiO_2_ upon performing reduction of metal ions at their surface [335]. The resulting CNHSs thus consisted of rutile TiO_2_ nanorods decorated with a dense population of tiny metal patches. Other syntheses leveraging on the thermolytic decomposition of metal complexes over oxide nanorod seeds in surfactant mixtures under reducing conditions resulted in Ag–TiO_2_ CNHSs with similar architectures, composed of multiply metal-decorated oxide nanorods [336,337,338] (Figure 10a).

Photocatalytic metal-ion reduction has also been exploited as a chemical pathway to sustain heterogeneous nucleation. As a general trend of such cases, one single metal domain was constructed in proximity of either termination of the nanorods that were used as seeds. For example, Ag-functionalized ZnO nanorods were obtained by a UV-driven photocatalytic reduction of AgNO_3_ reduction over ZnO nanorod seeds in the ethanol media [304]. Due to the lability of the pristine acetate anions adsorbed on the ZnO seeds (which were pre-synthesized upon alkaline hydrolysis of zinc acetate in alcohol), metallic Ag patches could readily deposit onto the nanorods, where they catalysed further Ag^+^ ion reduction at the surface of the initially nucleated domains, thus avoiding undesired homogenous nucleation in the liquid mixture. Good lattice-matching conditions at specific regions of the seeds were presumed to facilitate the establishment of such growth mode, even though a redistribution of photogenerated conduction electrons driven by an intrinsic internal dipole moment could also be invoked to play a role [281,310,314,339]. In another account, UV-driven photocatalytic reduction of AgNO_3_ in the presence of surfactant-protected TiO_2_ nanorods in a toluene solution containing OLAM and 1-hexadecanol led to the initial formation of heterostructures composed of multiple tiny Ag patches attached to each seed [340]. Upon prolonged irradiation, an intraparticle ripening process drove progressive hole-driven dissolution of the smaller Ag particles and reduction of the as-released Ag^+^ species onto a single (the largest and most stable) Ag domain, where the excess photogenerated electrons shuttled through the nanorods tended to localize (Figure 10b,c). This process was reminiscent of the earlier discovered mechanism underlying the conversion of double-tipped Au-CdSe-Au dumbbell-like CNHSs to their single-tipped Au-CdSe matchstick-like counterparts [281].

Finally, syntheses of anisotropic-shaped CNHSs based on a reverse seeding scheme, where metal CNCs are used as the starting substrates, have also been devised. In one reported study, manipulation of TiCl_3_ hydrolysis in aqueous media by regulation of pH allowed installing large TiO_2_ heads at the extremities of cetyltrimethylammonium bromide-(CTAB)-capped Au nanorods [341]. The TiO_2_ domains grew to a point at which they covered a fraction of the longitudinal facets at the nanorod terminal part (Figure 10d–k). Au-ZnO CNHSs, composed of a ZnO nanopyramid section decorated with a single Au domain at its basal side, were controllably synthesized in OLAM-based liquid mixtures by thermal decomposition of zinc acetate over Au CNCs [342,343] (Figure 10l). Another report documented the formation of Au–Fe_x_O_y_ CNHSs, composed of a spinel-phase Fe_x_O_y_ nanorod portion adjoined to a spherical Au@Fe_x_O_y_ core@shell domain on its longitudinal facets [344] (Figure 10m).

According to the developed protocol [344], spherical Au CNCs were first induced to react with Fe(CO)_5_ in ternary ODE-diluted mixture of surfactants, namely OLAM, OLAC and dodecyldimethylammonium bromide (DDAB), at 300 °C. In the early heating stages, Fe_x_O_y_ deposited on the Au seeds as a thin a thin, discontinuous layer. After protracted annealing, as a consequence of the strain developing at the Au/Fe_x_O_y_ heterojunction and of the site-preferential coordination of DDAB to Fe_x_O_y_, the initially symmetric evolution of Fe_x_O_y_ was interrupted, switching to an anisotropic evolution mode, whereby a Fe_x_O_y_ rod-like section evolved tangential to each pre-existing Au@Fe_x_O_y_ core@shell substrate (Figure 10m). As a secondary proof for the envisaged mechanism of growth of the resulting Au@Fe_x_O_y_–decorated Fe_x_O_y_ nanorod CNHSs, Au could be selectively etched away from the heterostructures, leaving composite structures individually made of one Fe_x_O_y_ capsule connected to one Fe_x_O_y_ rod-shaped section (Figure 10n).

Unconventional heterostructures with branch-type connectivity deserve to be mentioned. Ag-ZnO multipod CNHSs were obtained by epitaxial development of ZnO rod-shaped domains from the (111)-type facets exposed by Ag seeds with a truncated cube shape [345] (Figure 10o). Critical to guaranteeing the synthesis outcome was considered to be facet-dependent accessibility of the Ag seeds, as governed by selective surface binding of the PVP polymer selected as capping agents to assist ZnO in the specified alkaline media.

Analogous multiarmed architectures, composed of a Au_3_Cu core from which multiple ZnO branches departed out, were obtained in one-pot approach by reaction of earlier-generated Au_3_Cu CNCs with zinc acetate in OLAM/benzyl alcohol environment [207].

To further elaborate on a CNHS architecture, it can be considered that selected domains belonging to a preformed heterostructure with a starting binary composition may, in principle, be exploitable as reactive points onto which an additional material domain may be directed to deposit, thus leading to a corresponding heterostructure with a ternary composition [346,347,348]. Thus, a rationally designed reaction scheme involving multiple seeding steps can be regarded as a natural route to enhance the compositional richness and structural complexity of CNHSs.

Successful attempts have been made to realize the synthetic goals highlighted above. In one relevant study, Pt–tipped Cd@CdS core@shell nanorod seeds were used to induce the otherwise unfavorable deposition of Co onto the Pt termini, thus affording Pt@Co–CdSe@CdS–Pt@Co CNHSs with dumbbell-shaped profile and Co@Pt heads. These heterostructures could be modified further into Pt@Co_x_O_y_–CdSe@CdS–Pt@Co_x_O_y_ CNHSs with Pt@Co_x_O_y_ yolk@shell heads via selective oxidation of the Co components [346] (Figure 11a,b). More recently, a conceptually similar synthetic scheme has been applied to construct matchstick-shaped Fe_3_O_4_@Au–Zn*_x_*Cd_1__−_*_x_*S CNHSs starting from Au–tipped Zn*_x_*Cd_1__−_*_x_*S nanorods or Ag_2_S–tipped Zn*_x_*Cd_1__−_*_x_*S nanorods, where the Au or Ag_2_S tips served to promote regioselective nucleation and growth of Fe_3_O_4_ heads thereon [347]. In another protocol [348], a photodeposition reaction that involved an intraparticle metal ripening process [281], was exploited to accommodate a single Au nanocrystal onto one of the four arms of pre-existing Cd@CdS core@shell tetrapod-like seeds, leading to CNHSs made of asymmetrically Au-tipped CdSe@CdS tetrapod skeletons (Scheme 4b). Subsequently, thermolysis of Co_2_(CO)_8_ in the presence of the Au-CdSe@CdS tetrapod-based heterostructures and polystyrene ligands equipped with carboxylic-acid terminals in 1,2,4-trichlorobenzene at T = 140 °C allowed coating the previously installed Au domains with a Co layer. Finally, after facile Co oxidation, the heterostructures were converted to corresponding CdSe@CdS core@shell tetrapod CMHSs asymmetrically decorated with a single Au@Co/Co_x_O_y_ core@shell head at one apex (Figure 11c).

In most situations, the intimate connection between semiconductor and metal domains led to electronic coupling and/or to charge-density redistribution, which indirectly manifested as photoluminescence quenching or altered plasmonic absorption, along with enhanced exciton-dissociating capabilities [34,36,291,341,349]. Localized defect states at the concerned heterointerfaces were additionally proposed to explain the significant deviations in the optoelectronic behavior observed for such CNHSs [34,36,291,325,326,349,350]. Depending on the liquid environment, the presence of hole or electron scavengers, and the illumination conditions, the metal domains could either act as a sink for the photoexcited electrons generated in the semiconductor section or facilitate their transfer to the liquid medium. This mechanism could be rationally exploited to manipulate charge-storing or photocatalyzed reducing capabilities of CNHSs [34,36,291,328,331,333,341,349,351,352]. The magnetic properties were also modified upon the formation of heterojunctions [308,328,333,334]. Finally, it is worth highlighting that tip functionalization with magnetic metal domains has been proven to be an effective approach to promoting the self-organization of CNHSs into inorganic assemblies by leveraging on head-to-head magnetic attraction [346,347,348].

#### 4.2.2. Surfactant-Controlled Facet-Selective Heterogeneous Nucleation

For many materials combinations, detailed insights into the conditions that guaranteed the evolution of CNHSs with nonequivalent configurations has provided indirect confirmation for the operation of the frequently credited mechanism of facet-selective binding of organic stabilizers as the key process that governs the site-dependent accessibility and chemical reactivity of shape-tailored nanocrystal seeds (Scheme 4). Examples of CNHSs that were elaborated by leveraging on this mechanism are shown in Figure 12.

The unambiguous impact of the growth environment on the possible locations of an anisotropically shaped seed on which secondary material domains can be deposited was assessed for Co–TiO_2_ CNHSs composed of Co-functionalized TiO_2_ nanorods [315]. These heterostructures were prepared upon decomposition of Co_2_(CO)_8_ in the presence of OLAC-capped TiO_2_ nanorods in an ODE-diluted mixture of octanoic acid (OCAC) and OLAM at 250–280 °C, followed by rapid injection of OLAC (Figure 12a,b). It was found that judicious manipulation of the temporal variation of the OCAC and OLAM concentrations in the reaction environment served to regulate the accessibility of the cobalt precursor to the different facets exposed by the TiO_2_ nanorod seeds; the swift addition of OLAC allowed quenching heterogeneous nucleation of Co at the desired stage, without blocking growth of the earlier-formed Co nanocrystals. Such level of control allowed Co evolution over the TiO_2_ nanorods to switch from a tip-preferential to a nonselective deposition regime, in which metal domains nucleated and grew either on the apexes only, or randomly along the longitudinal sidewalls of the seeds, respectively (Scheme 4a, path one vs. path three). According to detailed HRTEM analyses within the framework of the CSLT theory [267], both types of Co–TiO_2_ CNHS configurations were estimated to be nearly equivalent based on the degree of unrelaxed interfacial strain that should be tolerated at the various types of heterojunctions attained. Hence, preferential Co overgrowth on selected families of facets of the TiO_2_ nanorod seeds was interpreted as a process that allowed compensating for the increase in the total surface energy of the system, which would otherwise be caused by depletion of the passivating ligand population on those surface locations (such condition would set in below a critical surfactant concentration in the bulk solution). Finally, it is interesting to recall that these Co-TiO_2_ CNHSs exhibited an anomalous modification in the magnetic anisotropy of the nanoscale Co domains, which was attributed to proximity effects enabled by electronic communication through the rather extended heterointerfaces [315].

Another report confirmed that a surfactant-controlled heterogeneous nucleation-growth mechanism governed the formation of exotic bi-magnetic CNHSs composed of a γ-Fe_2_O_3_ tetrapod-based skeleton connected to randomly distributed Co satellites [318] (Scheme 4b, path three). In these ramified nanoheterostructures (Figure 12c–f), the FiM-FM exchange coupling that set in between Co and γ-Fe_2_O_3_ at the numerous heterointerfaces was manifested into a diversity of exclusive magnetic properties, including noticeable exchange bias, increased saturated magnetization and coercivity, and enhanced thermal stability of the magnetization [318]. More recently, in the reversed material configuration of Fe_3_O_4_-decorated Co nanorods (Scheme 4a, path three), the hard magnetic properties of Co were found to be preserved and dominate the overall magnetic behaviour of the CNHSs [319].

Finally, an interesting one-pot synthetic approach to CNHSs composed of multiply Co-decorated CoO tetrapods has also been described [353]. The synthesis relied on the reaction of Co(acac)_2_ with 1,2-dodecanediol in ODE. The mixture was heated for different periods at 200 °C and 320 °C, respectively. The Co-CoO CNHSs were found to evolve across a sequence of kinetically self-regulated nucleation-growth phases that occur at distinct times, corresponding to the different temperatures at which the reaction mixture was annealed (see also Section 4.1.3). In the early stages, Co(acac)_2_ was decomposed, sustaining the formation of the CoO tetrapods; subsequently, the reduction of Co(acac)_2_ by 1,2-dodecanediol led to heterogeneous deposition of multiple Co domains on the weakly surfactant-passivated CoO tetrapod seeds.

## 5. Conclusions

Design and realization of modular constructs that incorporate epitaxially joint metal and metal-oxide nanocrystal domains belongs to a frontier area of nanochemistry research, where a wealth of fundamental and practical knowledge has been gained over the past years. The solution-phase fabrication is of CNHSs is a heavily challenging enterprise, which requires integrating the capability to tailor the lattice structure and geometry of the targeted material modules with the comprehension of the thermodynamic factors and kinetic processes that may underlie their sequential arrangement in space via liquid-phase heteroepitaxy. Progress reported so far suggests that elevating the level of configurational sophistication and regioselectivity in CNHS construction could be reachable by consolidating the understanding of the microscopic mechanisms by which CNHSs may evolve.

To date, the potential of metal/metal-oxide based CNHSs for feeding current and novel technologies remains still largely unmet due to the modest degree of synthetic accuracy and reproducibility with which these nanoheterostructures (and colloidal nanocrystals in general), hence their properties, can be delivered beyond the laboratory production scale to serve defined purposes. The hunt for multifunctionality often contrasts with partial (often, unavoidable) degradation of the expected properties of the otherwise bare modular constituents. In the vast majority of cases, such drawback is likely to derive from unfavourable alterations in electronic structure and/or from the attainment of defective heterointerfaces. As a matter of fact, it remains hard to explicitly distinguish between trivial proximity effects and the genuine manifestation of novel properties arising from electronic contact and exchange-coupling mechanisms. In this respect, heavier support from theoretical studies, which are rather rare, would be beneficial in an effort to decipher the actual impacts of heterojunctions on the ultimate physical-chemical behaviour of CNHSs.

In a prospective, it is conceivable that further advances in the design and construction of CNHSs leveraging on enhanced synthetic ingenuity will open access to unprecedented insights into the chemistry and physics of colloidal heterostructures and facilitate the transfer of their properties and functionalities into technologies relevant to many research domains of optoelectronics, energy production and storage, (photo)catalysis, and biomedicine.
